# A meta-analysis on individual differences in primary emotional systems and Big Five personality traits

**DOI:** 10.1038/s41598-021-84366-8

**Published:** 2021-04-02

**Authors:** Davide Marengo, Kenneth L. Davis, Gökçe Özkarar Gradwohl, Christian Montag

**Affiliations:** 1 Department of Psychology, University of Turin, Turin, Italy; 2 Pegasus International, Inc, Greensboro, NC USA; 3 Çınar Psychotherapy Center, Istanbul, Turkey; 4 Department of Molecular Psychology, Institute for Psychology and Education, Ulm University, Helmholtzstraße 8/1, 89081 Ulm, Germany

**Keywords:** Psychology, Human behaviour

## Abstract

The Affective Neuroscience Personality Scales (ANPS) were constructed as a self-report assessment to measure individual differences in Jaak Panksepp’s cross-species primary emotional systems: SEEKING, PLAY, CARE (positive emotions) and FEAR, SADNESS, ANGER (negative emotions). Beginning with the first published work on the ANPS in 2003, individual differences on the ANPS measures of these six primary emotional systems have been consistently linked to Big Five personality traits. From a theoretical perspective, these primary emotional systems arising from subcortical regions, shed light on the nature of the Big Five personality traits from an evolutionary perspective, because each of these primary emotional systems represent a tool for survival endowing mammalian species with inherited behavioral programs to react appropriately to complex environments. The present work revisited 21 available samples where both ANPS and Big Five measures have been administered. Our meta-analytical analysis provides solid evidence that high SEEKING relates to high Openness to Experience, high PLAY to high Extraversion, high CARE/low ANGER to high Agreeableness and high FEAR/SADNESS/ANGER to high Neuroticism. This seems to be true regardless of the ANPS inventory chosen, although much more work is needed in this area. Associations between primary emotional systems and Conscientiousness were in the lower effect size area across all six primary emotions, thereby supporting the idea that Conscientiousness rather seems to be less directly related with the subcortical primary emotions and likely is the most cognitive/cortical personality construct out of the Big Five. In sum, the present work underlines the idea that individual differences in primary emotional systems represent evolutionarily ancient foundations of human personality, given their a) meaningful links to the prominent Big Five model and b) their origins lying in subcortical areas of the human brain.

## Introduction

Personality could be described as relatively stable motivational, emotional, cognitive and behavioral traits of a person impacting on many important life variables ranging from health behavior, longevity, job performance to vulnerability for affective disorders (for an overview see^1^). Of note, defining personality still remains a highly controversial topic, as a recent discussion paper shows^2^. Beyond clinical and lexical approaches to study human personality (e.g.^3–5^), many theories attempt to explain the biological basis of personality, including Gray’s reinforcement sensitivity theory, Cloninger’s biosocial theory of personality or Eysenck’s PEN model^6^.

A relatively new addition to biopsychological-oriented theories of personality has been Panksepp’s Affective Neuroscience Theory^7,8^. By means of many approaches, including electrical brain stimulation, lesion studies and pharmacological challenges, Panksepp carved out seven primary emotional systems, all being homologously conserved across the mammalian brain^9^. The homologous conservation of such primary emotional systems across different mammalian species speaks for the idea that such systems endow their carriers with evolutionary advantages—each primary emotional system can be seen as a tool for survival. On the positive side of emotions Panksepp mapped the neuroanatomy and biochemistry underlying the primary emotional systems he labeled SEEKING, LUST, CARE, and PLAY, whereas on the negative side ANGER, FEAR and SADNESS have been similarly illuminated by Panksepp’s research group. In short, these systems provide evolutionary advantages, as they endow mammals with energy to seek for food or a partner (SEEKING), reproduce and transfer one’s own genome (LUST), secure the upbringing of their offspring (CARE) and a system for learning social competencies and motoric skills (PLAY). The negative primary emotional systems help to bring mammals out of the danger zone with a fight system to guard significant resources including one’s own offspring (RAGE/ANGER), a fight/flight/freezing program to cope with physical dangers (FEAR), and maintain protective social contact and avoid separation from caregivers and loved ones resulting in separation distress (SADNESS). For readers interested in the brain structures and neurotransmitter/neuropeptides underlying these primary-process emotional action systems we refer to Panksepp’s seminal work called Affective Neuroscience^9^ or to a more recent summary^10^. Also, a detailed introduction into principles of Pankseppian Affective Neuroscience is beyond this brief article, and the interested reader could find an overview in Davis and Montag^11^.

Based on the Affective Neuroscience Theory^7^, the *Affective Neuroscience Personality Scales* (ANPS) assess six of the seven primary emotional systems via self-report, namely SEEKING, ANGER, FEAR, CARE, SADNESS and PLAY. LUST has not been included in the ANPS self-report inventory, given the chance that filling in items on one’s own sexuality could elicit socially desirable answers with a negative spill-over effect on the answers given on the remaining items of the questionnaire.

Accepting the limitations that subcortically-based primary emotions are pre-propositional and that a language-based assessment must necessarily operate in a more tertiary cerebral space, when writing the ANPS items the goal was to ask questions that addressed personal emotional feelings and their behavior elaborations as directly as possible^7^. In contrast to most personality assessments, items were avoided that required making more cognitive judgments such as “I prefer spending time at a popular beach to an isolated nature reserve” or social comparisons such as “I am more energetic than other people.” More “projective” items such as “I believe that …” were also avoided. The intention was to position people in affective space.

The ANPS illustrates that Pankseppian primary emotional systems do not operate at same strength level in all mammals (including sapiens). Instead, although these primary emotional systems operate in all mammals, they do this to varying energy levels. Montag & Panksepp^12^, [p. 10] discuss in detail the evolutionary advantage, from a species perspective, of the fluctuation selection concept. They summed up: “The term fluctuation selection (see also Nettle, 2009^13^, p. 64) illustrates that not always is the same trait associated with higher survival, but rather that the trait that is best adapted varies with environmental changes.” Hence, it is good from a species perspective to always have a reservoir of different operating primary emotional systems in the genomic pool, as some of one’s own species will likely be able to adapt to unforeseen changes in the environment.

In the original work by Davis and colleagues^7^, [p. 60] the ANPS was validated against a reduced set of Goldberg’s Big Five personality markers, which measured Extraversion, Agreeableness, Conscientiousness, Emotional Stability (opposite of Neuroticism), and Openness to Experience. The Big Five were seen to represent a theory-free and widely accepted approach to personality assessment. Further, it was hypothesized that studying the lexically derived Big Five personality traits in relation to the Affective Neuroscience Theory, could shed light on evolutionary aspects of the Big Five, namely, which evolutionary ancient brain systems are linked to the Big Five. It could be observed in this early work that SEEKING and Openness to Experience were strongly positively related and the same was true for PLAY and Extraversion. Beyond that, high CARE and low ANGER have been robustly linked to higher Agreeableness, while lower FEAR/ANGER/SADNESS have been linked to higher Emotional Stability. Subsequently, similar observations also have been observed in German, Chinese, French, Italian, Spanish, Turkish and Serbian samples^12,14–18^.

To sum up our points so far: Primary-process emotional systems are phylogenetically ancient brain systems that arise from subcortical brain regions. In contrast, the Big Five factors have been derived from the statistical analysis of language. This lexical approach speaks in general for a more cerebrally-oriented approach to understanding personality. Viewing the relationship between the ANPS and Big Five scales from an evolutionary brain development framework could begin the process of understanding individual differences in Panksepp’s primary emotional action systems as bottom-up neurobiological underpinnings of the Big Five personality traits (for a more extensive discussion, see Davis and Panksepp^3^, and more recently, Montag with Davis^19^).

We acknowledge that the present meta-analysis provides only correlational evidence for this hypothesis. However, additional supportive evidence comes from clinical research – especially for the SADNESS scale as perhaps the most clinically relevant of the ANPS scales. Consistent with affective neuroscience predictions^20^, Montag and colleagues^21^ reported that in a group of 55 clinical inpatients being treated for depression the strongest correlation between the Beck Depression Inventory-II and the ANPS scales was with the SADNESS scale (r = 0.53, *p* < 0.001). This finding that the ANPS SADNESS scale was related to depression was replicated by Fuchshuber and colleagues^22^ who applied path analysis to a large sample (n = 616 including 147 diagnosed with depression) showing that depressive symptoms as measured by the Brief Symptom Inventory were again most strongly predicted by the ANPS SADNESS scale (beta = 0.52).

Further evidence that ANPS scales are associated with biologically-based psychopathology comes from a pair of studies on bipolar disorder. Savitz and colleaguesl^23,24^ studied 300 individuals from 47 families including five subgroups: 58 Bipolar I cases, 27 Bipolar II cases, 58 with recurring major depression, 45 with a single depressive episode, and 88 unaffected family members. As expected, in the first study^23^, they found that Bipolar I diagnosed individuals scored the highest on the ANPS SADNESS scale and significantly higher than unaffected relatives. In the second study with four cases dropping out^24^, Bipolar II diagnosed individuals predictably scored highest on the ANPS ANGER scale and significantly higher than unaffected relatives. In both studies, ANPS SADNESS and ANGER score decreases in each of the family subgroups were consistent with their pathological severity. Lastly, the SADNESS scale has also been anatomically linked to the amygdala resting state activity in a functional connectivity analysis^25^, which is consistent with Affective Neuroscience theory (AN theory)^9^, [pp. 267–268].

The evidence linking the ANPS to Panksepp’s primary emotions and explicitly showing how the activity of these primary emotional systems function as a bottom up subcortical foundation of the Big Five personality traits are in early stages. However, we believe these hypotheses to be highly relevant to an understanding of the biological basis of personality and offer this ANPS-Big Five meta-analysis as a valuable step in that direction.

Since the initial publication of the ANPS in 2003, many studies have investigated the ANPS in the context of different Big Five versions (e.g. NEO-FFI, NEO-PI-R, BFI or TSDI). Moreover, different versions of the ANPS have been administered in these Big Five-ANPS works, namely, the 2003 original ANPS version and the ANPS 2.4^8^. Beyond that, several short forms exist: B-ANPS or ANPS-S. Recently, a short adjective based ANPS-AR has been published^26^. Please also note that the ANPS is available in many languages, including Spanish, French, German, Turkish, Norwegian, Italian, Polish, Portuguese, Brazilian Portuguese, Chinese, Japanese, Serbian, Persian and soon to be in Hungarian and Dutch. This all said, the present study aims to investigate the overall strength between ANPS-Big Five associations by conducting a meta-analysis on the available data stemming from studies conducted all over the world. Additionally, we explore the use of different assessments of Big Five personality traits as a source of heterogeneity in reported associations, focusing in particular on potential differences between studies using the NEO Inventories vs. alternative operationalizations of the Big Five model.

From the literature review, we expected that high SEEKING is associated with high Openness to Experience, high PLAY with high Extraversion, low ANGER and high CARE with high Agreeableness and high FEAR, SADNESS and ANGER with high Neuroticism. From correlation strength we expect ANGER to be less strongly associated with Neuroticism than the FEAR/SADNESS associations. Regarding SEEKING in the literature, associations with Extraversion turned out to be more heterogenous, but we nevertheless expected a positive association. Finally associations with Conscientiousness and primary emotions should be in the lower area due to Conscientiousness being the most cerebrally-focused personality dimension out of the Big Five personality measures.

## Method

### Literature search

In order to identify papers investigating the association between ANPS and Big Five personality traits, we followed PRISMA guidelines^27^ and implemented a study selection strategy based on predetermined eligibility criteria and involving literature searches in the Scopus, ISI Web of Science, and PubMed citation databases. In order to keep the search results as broad as possible, in querying the databases we looked for all papers mentioning use of ANPS instruments using the following strings of keywords: “affective neuroscience personality scale” OR “affective neuroscience personality scales”.

**Figure 1:**
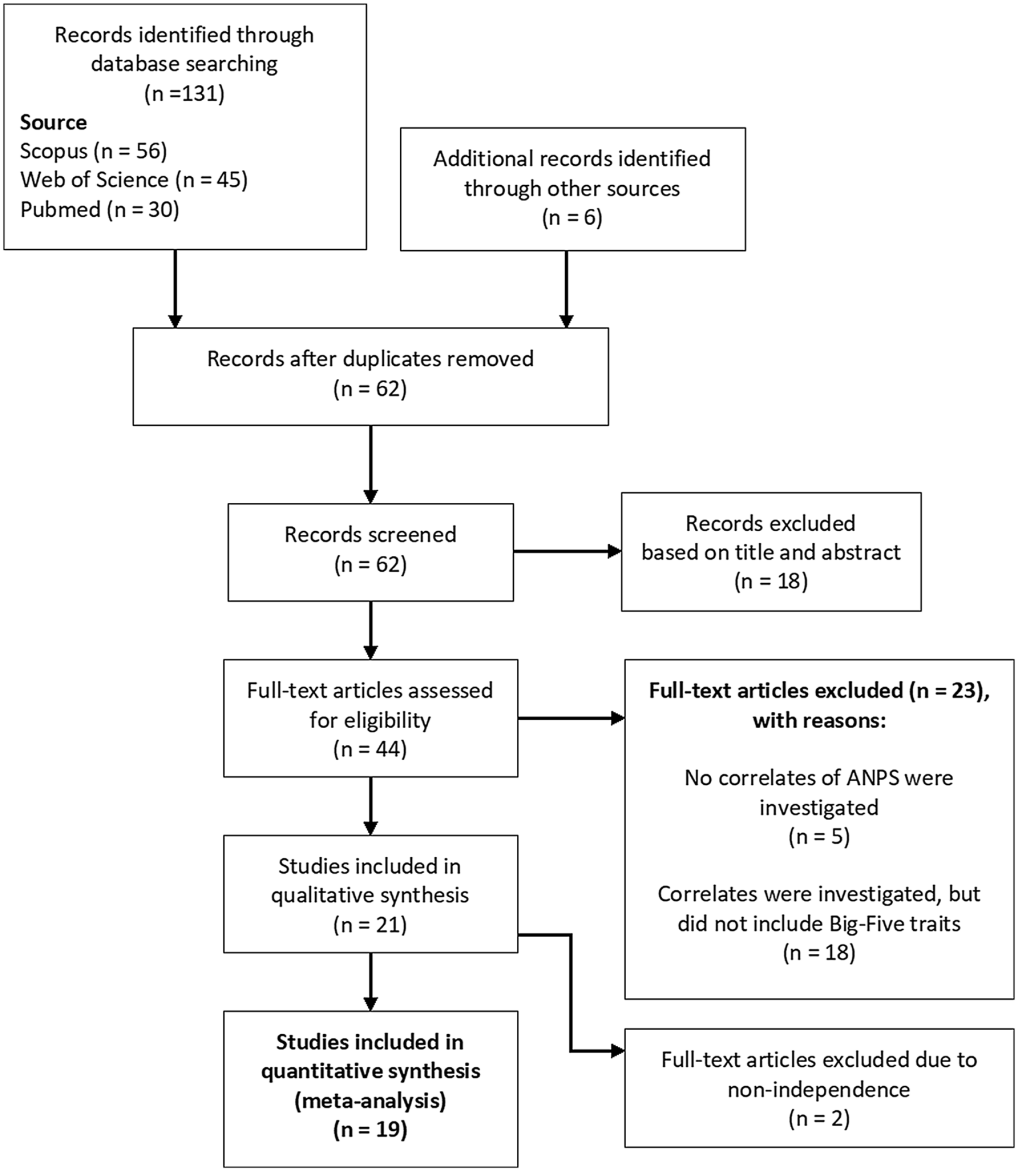
Flow chart of study selection.

The database search was finalized in April 2020. An additional search was performed by inspecting citations within publications identified as eligible to be included in this meta-analysis. A flowchart illustrating the selection process is shown in Fig. 1.

### Inclusion and exclusion criteria

Papers identified through database and reference searches were screened for the following inclusion criteria: 1. Studies had to include assessments of both ANPS and Big Five personality traits; 2. Studies had to provide information about the effect size of the association between ANPS and Big Five personality traits.

Exclusion criteria were the following: 1. Studies were excluded from quantitative analyses if we could not retrieve effect size information by inspecting the paper, and this information could not be obtained from the authors; 2. Non-independence of collected data. Studies were considered as non-independent when fulfilling the following criteria: 1. Studies were performed on samples including the same group of participants, and 2. Effect-sizes were computed between the same ANPS and Big Five measures. When two or more studies were found to be non-independent according to these criteria, we selected the study performed on the largest sample for the purpose of inclusion in the quantitative analyses. When a complete overlap between samples was found, we selected the earliest study.

### Strategy of analyses

For the purpose of this study, we use Pearson’s correlation coefficients as the effect-size of choice for representing the relationship between ANPS and Big Five personality traits. In the case we could not collect Pearson’s correlations by inspecting the paper (e.g., examined correlations were not reported in full in the results section), we contacted the authors of the study and asked them to provide us with missing correlations.

We conducted a separate meta-analysis for each combination of ANPS and Big Five trait, resulting in a total of 30 distinct meta-analyses (6 ANPS × 5 Big Five traits). We performed the meta-analyses using a random-effects model, as we expected significant heterogeneity in effect-sizes due the varying characteristics of questionnaires used to assess Big Five personality, as well as due to the diversity of cultural and demographic characteristics of the samples. For the purpose of meta-analytical computations, we decided not to transform correlations into Fisher’s z scores because this transformation leads to an overestimation of associations when compared with the original correlation metric^28^.

Heterogeneity of effect-sizes was determined using the following statistics: the Q test of heterogeneity, the *Ƭ*^2^ and *Ƭ* statistic statistics (i.e., between-study variance and standard deviation of effect-sizes), and the *I*^*2*^ statistic representing the proportion of variance in observed effects due to true heterogeneity (as opposed to random sampling error). In discussing emerging meta-analytic correlations, we refer to Cohen’s well-known classification of correlation effect-sizes, and distinguish between small (0.10 ≤ r < 0.30), medium (0.30 ≤ r < 0.50), and strong correlations (r ≥ 0.50).

Next, we evaluate the impact of the different operationalizations of the Big Five model as a source of heterogeneity. More in detail, we use the Q test for heterogeneity to compare correlations between ANPS and Big Five traits emerging from studies employing NEO inventories to assess the Big Five traits, and studies employing other Big Five operationalizations. Please note that because we expected that relevant differences might exist across these groups in terms of cultural and demographic characteristics of recruited samples, in performing the Q tests we do not assume homogeneity of variances of effect sizes across these groups (i.e., we use separate estimates of *Ƭ*^2^, as opposed to a pooled estimate).

Finally, we inspected results for potential publication bias by investigating the existence of asymmetry in the funnel plots visualizing the association between studies’ effect sizes and their relative standard error. For detecting asymmetry of the funnel plots, estimation was performed on transformed effect sizes (Fisher’s z transformation). Symmetry of the funnel plot was determined using Egger’s intercept test^29^. If the Egger’s test detected a significant asymmetry in the funnel plot, we used Duval and Tweedie's Trim and Fill procedure^30^ to impute effect-size data for potentially missing studies and compute the unbiased meta-analytical correlation. All analyses were performed using Comprehensive Meta-analysis 3^31^.

## Results

### Overview of included studies

In total, we identified 21 documents including data on both ANPS and Big Five personality scales. After inspection for non-independence, we found n = 8 document included non-independent studies, i.e., studies that were performed on samples including overlapping participants assessed using the same set of ANPS and/or Big Five measures^7,8,12,17,32–35^. The study by Montag and Panksepp^12^ included information about German and Chinese samples that overlapped with two samples examined in Sindermann and colleagues^35^. To resolve the issue, we selected the largest German sample (i.e., the sample examined in^12^), and the largest Chinese sample (i.e., the sample examined in^35^). Next, we found a (likely) overlap in the German samples examined in studies by Özkarar-Gradwohl and colleagues^32^, Plieger and colleagues^33^, and Reuter and colleagues^34^, which we resolved by selecting the study with the largest sample^34^. However, while all participants from the sample in Reuter and colleagues^34^ were assessed using paper and pencil questionnaires, a subset (N = 71) of the German sample examined in^32^ was assessed via online questionnaires (and on the Big Five side the B5S was administered), therefore showing no overlap. This subset was retained in the dataset for the purpose of performing the meta-analytical calculations. We also found two papers by Özkarar-Gradwohl and colleagues^17,32^ which included analyses performed on the same sample of Turkish participants; in this case, we selected the earliest study^17^ for inclusion in the meta-analysis. Finally, we found that two studies by Davis and colleagues^7^ and Davis and Panksepp^8^ presented results from the same sample; again, the earliest study^7^ was selected for inclusion.

Eventually, we ended up with 19 documents, including data on both ANPS and Big Five personality collected on 21 independent samples, resulting in 612 distinct effect sizes representing the association between ANPS and Big Five personality scales: For all selected studies we were able to retrieve effect-size information about the association between all the ANPS behavioral scales (ANGER, CARE, FEAR, PLAY, SADNESS, and SEEKING) and Big Five traits (Agreeableness, Conscientiousness, Extraversion, Neuroticism, and Openness), except for a study by Yu^36^, which only reported information about ANPS scales and two Big Five traits (Agreeableness, Consciousness). Characteristics of selected studies are reported in Table 1.

**Table 1:** Characteristics of studies included in the meta-analysis.

Study	Sample characteristics		Self-report assessment		
	Country	Type of Sample	N	ANPS	Big Five
Abella et al., 2011^14^	Spain	General population	397	ANPS—112	NEO-FFI-R
Barrett et al., 2010^38^	USA	General population	226	ANPS—110	BFI
Barrett et al., 2013^46^	USA	General population	1644	BANPS	BFI
Cwojdzińska and Rybakowski, 2016^47^	Poland	General population	78	ANPS—112	NEO-FFI
Davis et al., 2003^7^	USA	General population	171	ANPS—110	B5S
Giacolini et al., 2017—Study 1^15^	Italy	Clinical sample	180	ANPS—112	BFI
Giacolini et al., 2017—Study 2^15^	Italy	General population	523	ANPS—112	BFI
Hiebler-Ragger et al., 2018^48^	Austria	General population	167	ANPS—110	BFI
Montag and Davis, 2018^26^	Germany	General population	182	ANPS—110	Big Five short-scale
Montag and Panksepp, 2017^12^	Germany	General population	687	ANPS—110	NEO-FFI
Montag et al., 2019^16^	Serbia	General population	340	ANPS—112	NEO-PI-R
Montag et al., 2020^49^	Germany	General population	850	ANPS—110	BFI
Özkarar-Gradwohl et al., 2014^17^	Turkey	General population	327	ANPS—110	B5S
Özkarar-Gradwohl et al., 2018—Study 1^32^	Germany	General population	71	ANPS—110	B5S
Özkarar-Gradwohl et al., 2018—Study 2^32^	Japan	General population	353	ANPS—112	B5S
Pahlavan et al., 2008^18^	France	General population	412	ANPS—110	B5S
Reuter et al., 2017^34^	Germany	General population	1837	ANPS—110	NEO-FFI
Sindermann et al., 2018^37^	Germany	Clinical sample	52	ANPS—110	NEO-FFI
Sindermann, Luo, et al., 2018^35^	China	General population	615	ANPS—112	Big Five short-scale
Yu, 2018^36^	Hong-Kong	General population	157–159^a^	ANPS—110	IPIP Big Five scales ^b^
Yu, 2016^50^	Hong-Kong	General population	655–668^a^	ANPS—110	NEO-FFI

*ANPS* Affective Neuroscience Personality Scales, *BANPS* Brief Affective Neuroscience Personality Scales, *B5S* Big Five Scales, *BFI* Big Five Inventory, *NEO-FFI* NEO Five-Factor Inventory, *NEO Five-Factor Inventory-R* NEO Five-Factor Inventory-Revised.

^a^In these studies, sample size varied based on the specific combination of ANPS and Big Five scales examined.

^b^Only the Agreeableness and Conscientiousness traits were assessed in the study.

Mean sample size was 473.29, ranging from 52^37^ to 1,837 participants^34^, with an overall combined sample size of 9,939 individuals. The majority of samples were recruited among the general population (n = 19), while a minority (n = 2) were clinical samples. Included studies varied in terms of nationality: the majority of studies were performed on samples recruited in Germany (n = 6), followed by USA (n = 3), Hong-Kong (n = 2), Italy (n = 2), Austria (n = 1), China (n = 1), France (n = 1), Japan (n = 1), Poland (n = 1), Serbia (n = 1), Spain (n = 1), and Turkey (n = 1).

In the selected studies, ANPS scales were assessed using the original ANPS version (n = 13)^7^, the revised ANPS 2.4 (n = 7)^8^, and the Brief ANPS (n = 1)^38^. In selected studies, Big Five traits were assessed using either original or revised versions of the following Big Five personality questionnaires: the Big Five Inventory (n = 6; BFI)^39^ , NEO-Five Factor Inventory (n = 5; NEO-FFI; n = 1 NEO-FFI-R)^40^, the NEO Personality Inventory (n = 1; NEO PI-R)^40^, the Big Five Scales (n = 5; B5S)^7^, the Big Five short scales (n = 2)^41^, and Big Five scales from the Internet Personality Item Pool (n =1).

### Meta-analytic computations

#### Mean effect size

In order to establish the magnitude of associations between ANPS and Big Five personality scales, we conducted 30 separate meta-analyses, one for each combination of ANPS and Big Five scales. Because one study did not include information about all Big Five traits, the number of effect sizes included in the meta-analyses varied depending on the specific Big Five trait (Openness: n = 20; Conscientiousness: n = 21; Extraversion: n = 20; Agreeableness: n = 21; Neuroticism: n = 20).

**Figure 2:**
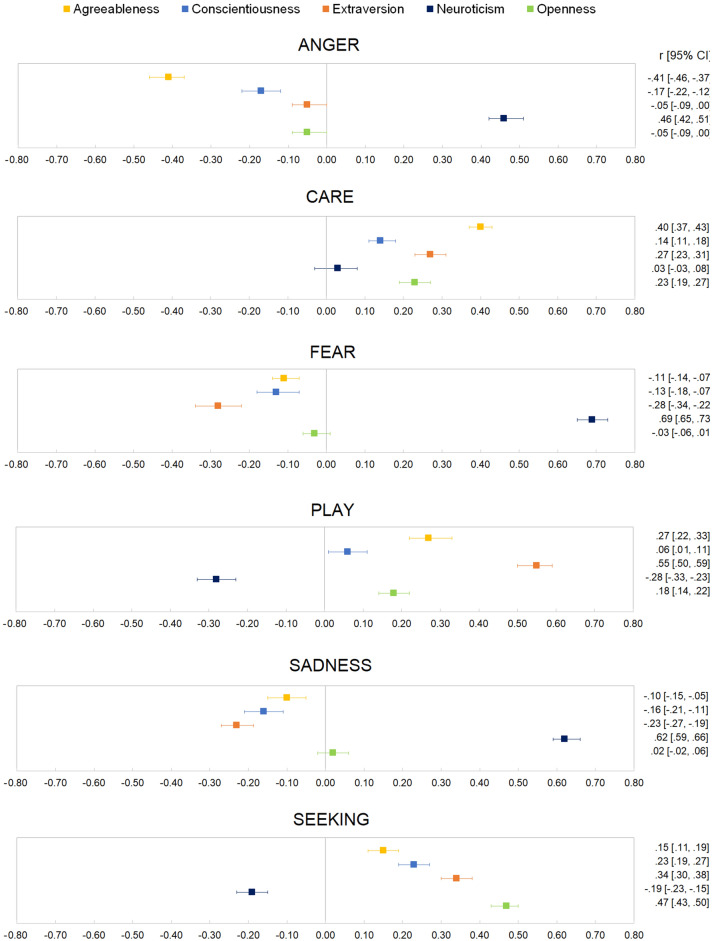
Forest-plots of meta-analytic correlations between ANPS and Big Five Personality scales.

For each combination of ANPS and Big Five personality scales, forest plots of meta-analytical correlations are presented in Fig. 2; the meta-analytic correlations are also presented in Table 2, alongside Q tests for heterogeneity, *Ƭ*^2^ and *I*^2^ statistics. Given the large number of examined study-level effect-sizes (n = 612), these are visualized in Table 2, and reported in the Supplementary material alongside the relative 95% confidence interval (Table S1).

**Table 2:** Meta-analytic correlations between ANPS and Big Five personality: Central tendency and heterogeneity statistics.

ANPS	Big Five	Correlation			Heterogeneity statistics					
		r	[95 % CI]		Q	df	p	i^2^	$\tau$^2^	$\tau$
ANGER	Agreeableness	− .41	− .46	− .37	142.38	20	< .01	85.95	0.01	0.12
	Conscientiousness	− .17	− .22	− .12	121.52	20	< .01	83.54	0.01	0.11
	Extraversion	− .05	− .09	.00	88.69	19	< .01	78.82	0.01	0.09
	Neuroticism	.46	.42	.51	139.06	19	< .01	86.34	0.01	0.12
	Openness	− .05	− .09	.00	80.33	19	< .01	76.35	0.01	0.08
CARE	Agreeableness	.40	.37	.43	49.12	20	< .01	59.28	< 0.01	0.06
	Conscientiousness	.14	.11	.18	57.36	20	< .01	65.12	< 0.01	0.07
	Extraversion	.27	.23	.31	90.25	19	< .01	78.95	0.01	0.09
	Neuroticism	.03	− .03	.08	134.33	19	< .01	85.86	0.01	0.12
	Openness	.23	.19	.27	76.13	19	< .01	75.04	0.01	0.08
FEAR	Agreeableness	− .11	− .14	− .07	59.53	20	< .01	66.05	< 0.01	0.07
	Conscientiousness	− .13	− .18	− .07	145.35	20	< .01	86.24	0.01	0.12
	Extraversion	− .28	− .34	− .22	171.65	19	< .01	88.93	0.02	0.13
	Neuroticism	.69	.65	.73	287.01	19	< .01	93.28	0.03	0.18
	Openness	− .03	− .06	.01	38.47	19	.01	50.61	< 0.01	0.05
PLAY	Agreeableness	.27	.22	.33	150.51	20	< .01	86.71	0.02	0.12
	Conscientiousness	.06	.01	.11	116.33	20	< .01	82.81	0.01	0.10
	Extraversion	.55	.50	.59	142.77	19	< .01	86.69	0.01	0.12
	Neuroticism	− .28	− .33	− .23	113.86	19	< .01	83.13	0.01	0.10
	Openness	.18	.14	.22	74.47	19	< .01	74.49	0.01	0.08
SADNESS	Agreeableness	− .10	− .15	− .05	117.65	20	< .01	83.00	0.01	0.11
	Conscientiousness	− .16	− .21	− .11	101.53	20	< .01	80.30	0.01	0.10
	Extraversion	− .23	− .27	− .19	74.58	19	< .01	74.52	0.01	0.08
	Neuroticism	.62	.59	.66	117.92	19	< .01	83.89	0.01	0.11
	Openness	.02	− .02	.06	57.65	19	< .01	67.04	< 0.01	0.07
SEEKING	Agreeableness	.15	.11	.19	74.26	20	< .01	73.07	0.01	0.08
	Conscientiousness	.23	.19	.27	73.42	20	< .01	72.76	0.01	0.08
	Extraversion	.34	.30	.38	74.49	19	< .01	74.49	0.01	0.08
	Neuroticism	− .19	− .23	− .15	61.57	19	< .01	69.14	0.01	0.07
	Openness	.47	.43	.50	97.88	19	< .01	80.59	0.01	0.10

Overall, we found the ANGER component of the ANPS showed a moderately positive meta-analytical correlation with Neuroticism (r = 0.46), a small negative correlation with Conscientiousness (r = − 0.17), and a moderate negative correlation with Agreeableness (r = − 0.41). The meta-analytical correlations for the associations between ANGER and Extraversion (r = − 0.05) and between ANGER and Openness size (r = − 0.05) were near zero.

Regarding the CARE component of the ANPS, findings showed small positive meta-analytical correlations with the Extraversion (r = 0.27), Openness (r = 0.23) and Conscientiousness (r = 0.14) traits, and a moderate correlation with the Agreeableness trait (r = 0.40). The meta-analytical correlation between CARE and Neuroticism was near zero (r = 0.03).

The FEAR component of the ANPS showed a strong positive meta-analytical correlation with Neuroticism (r = 0.69), and small negative meta-analytical correlations with the Extraversion (r = − 0.28), Conscientiousness (r = − 0.13), and Agreeableness (r = − 0.11) traits. The meta-analytical correlation between FEAR and Openness was near zero (r = − 0.03).

The PLAY component of the ANPS showed a strong positive meta-analytical correlation with Extraversion (r = 0.55), and small positive meta-analytical correlations with the Agreeableness (r = 0.27) and Openness (r = 0.18) traits. Additionally, a small negative meta-analytical correlation emerged between PLAY and Neuroticism (r = − 0.28). The meta-analytical correlation between the PLAY component and Conscientiousness was positive (r = 0.06), but negligible in size.

Concerning the SADNESS component of the ANPS, we found a strong positive meta-analytical correlation with Neuroticism (r = 0.62), and small negative meta-analytical correlations with Extraversion (r = − 0.23), Conscientiousness (r = − 0.16), and Agreeableness (r = − 0.10). The meta-analytical correlation between SADNESS and Openness was not significant (r = 0.02).

Regarding the SEEKING component of the ANPS, we found moderate positive meta-analytical correlations with Openness (r = 0.47) and Extraversion (r = 0.34), small positive meta-analytical correlations with Conscientiousness (r = 0.23) and Agreeableness (r = 0.15), and a small negative meta-analytical correlation with Neuroticism (r = − 0.19).

Looking at heterogeneity statistics, we found the results of Q tests were significant for all combinations of ANPS and Big Five traits, supporting the use of the random effect model to compute the meta-analytical correlations. Still, it is worthy to note that for all trait combinations, *Ƭ*^2^ ranged between < 0.01 and 0.03, indicating low between-study heterogeneity (i.e., between-study variance in effect-sizes). Additionally, for all combinations, observed dispersion of effect sizes was largely due to true heterogeneity, as opposed to sampling error (93.28 ≥ *I*^2^ ≥ 50.61).

Finally, we look at the impact of different operationalization of the Big Five model on meta-analytic correlations between ANPS and Big Five personality traits. Results of Q tests, as well as emerging meta-analytical correlations, are shown in Table 3. Meta-analytic correlations between PLAY and both Extraversion and Neuroticism, and between Neuroticism and both SADNESS and SEEKING, were significantly stronger in size among studies employing NEO inventories (i.e., NEO-FFI, NEO-FFI-R, and NEO-PI-R inventories) when compared with studies using different Big Five operationalizations (e.g., the BFI, B5S, Big Five Short Scales and IPIP assessments; combined group was contrasted). Remaining ANPS-Big Five correlations showed no significant differences across the two groups of studies.

**Table 3:** Meta-analytic correlations between ANPS and Big Five personality traits by Big Five operationalization.

ANPS	Big Five	Correlation		Heterogeneity test		
		NEO Inventories	Other	Q	df	P
ANGER	Agreeableness	− 0.47	− 0.38	3.22	1	0.07
	Conscientiousness	− 0.15	− 0.18	0.17	1	0.68
	Extraversion	− 0.11	0.01	3.31	1	0.07
	Neuroticism	0.42	0.49	1.94	1	0.16
	Openness	− 0.03	− 0.05	0.20	1	0.66
CARE	Agreeableness	0.39	0.40	0.01	1	0.91
	Conscientiousness	0.15	0.14	0.07	1	0.80
	Extraversion	0.31	0.25	1.83	1	0.18
	Neuroticism	0.02	0.03	0.01	1	0.91
	Openness	0.28	0.20	2.27	1	0.13
FEAR	Agreeableness	− 0.08	− 0.12	1.08	1	0.30
	Conscientiousness	− 0.11	− 0.13	0.09	1	0.76
	Extraversion	− 0.28	− 0.28	< 0.01	1	0.97
	Neuroticism	0.73	0.67	1.38	1	0.24
	Openness	− 0.01	− 0.03	0.33	1	0.57
PLAY	Agreeableness	0.20	0.31	2.56	1	0.11
	Conscientiousness	0.08	0.05	0.33	1	0.56
	Extraversion	0.64	0.49	31.24	1	< 0.01
	Neuroticism	− 0.35	− 0.24	4.89	1	0.03
	Openness	0.18	0.18	< 0.01	1	0.97
SADNESS	Agreeableness	− 0.07	− 0.12	0.72	1	0.40
	Conscientiousness	− 0.14	− 0.17	0.29	1	0.59
	Extraversion	− 0.23	− 0.23	0.01	1	0.93
	Neuroticism	0.67	0.60	5.74	1	0.02
	Openness	0.06	− 0.01	2.72	1	0.10
SEEKING	Agreeableness	0.10	0.17	2.13	1	0.14
	Conscientiousness	0.27	0.22	1.13	1	0.29
	Extraversion	0.38	0.32	2.83	1	0.09
	Neuroticism	− 0.24	− 0.16	5.30	1	0.02
	Openness	0.47	0.47	< 0.01	1	0.99

### Publication bias

We inspected the funnel plots of standard error versus the correlation (see Supplementary materials, Figures S1-S30). Egger's regression tests (Table 4) indicated no significant evidence of asymmetry in the funnel plot for all combinations of ANPS and Big Five scales, except for the association between the ANGER component of the ANPS and Neuroticism. In this case, because of this potential indication of publication bias, we used the Duval and Tweedie’s procedure to detect potential missing studies on each side of the funnel plot: the procedure detected no (n = 0) missing effect-sizes (i.e., studies) on the left side and right side of the funnel plot. Overall, this step of analysis showed that no significant indications of publication bias were present in the examined literature.

**Table 4:** Publication bias analyses: Egger's regression test for funnel plot asymmetry.

ANPS	Big Five	Intercept	95 % CI		t	df	p
ANGER	Agreeableness	− 0.04	− 2.48	3.31	0.30	19	0.38
	Conscientiousness	− 0.00	− 2.68	2.67	< 0.01	19	0.50
	Extraversion	0.70	− 1.72	3.13	0.61	18	0.27
	Neuroticism	2.46	− 0.33	5.26	1.85	18	0.04
	Openness	1.33	− 0.89	3.55	1.26	18	0.11
CARE	Agreeableness	− 0.68	− 2.35	0.99	0.86	19	0.20
	Conscientiousness	− 0.40	− 2.23	1.42	0.46	19	0.32
	Extraversion	− 0.53	− 2.97	1.91	0.45	18	0.33
	Neuroticism	0.63	− 2.35	3.61	0.45	18	0.33
	Openness	0.48	− 1.77	2.72	0.44	18	0.33
FEAR	Agreeableness	− 1.27	− 3.04	0.50	1.50	19	0.08
	Conscientiousness	0.12	− 2.80	3.05	0.09	19	0.46
	Extraversion	0.36	− 3.02	3.74	0.22	18	0.41
	Neuroticism	− 0.28	− 4.66	4.09	0.14	18	0.45
	Openness	0.72	− 0.84	2.28	0.97	18	0.17
PLAY	Agreeableness	− 1.33	− 4.24	1.58	0.96	19	0.17
	Conscientiousness	− 1.01	− 3.58	1.01	0.82	19	0.21
	Extraversion	− 0.80	− 3.86	2.27	0.55	18	0.30
	Neuroticism	0.99	− 1.72	3.70	0.77	18	0.23
	Openness	0.56	− 1.64	2.77	0.53	18	0.30
SADNESS	Agreeableness	0.82	− 1.79	3.42	0.66	19	0.26
	Conscientiousness	0.55	− 1.88	2.99	0.48	19	0.32
	Extraversion	1.27	− 0.87	3.41	1.25	18	0.11
	Neuroticism	− 1.66	− 4.34	1.02	1.30	18	0.10
	Openness	0.63	− 1.30	2.57	0.69	18	0.25
SEEKING	Agreeableness	− 0.52	− 2.60	1.56	0.52	19	0.30
	Conscientiousness	0.72	− 1.33	2.78	0.74	19	0.23
	Extraversion	0.24	− 1.99	2.47	0.23	18	0.41
	Neuroticism	1.11	− 0.84	3.07	1.20	18	0.12
	Openness	− 0.50	− 3.04	2.05	0.41	18	0.34

## Discussion

The aim of the present meta-analysis was to investigate how the Affective Neuroscience Personality Scales (ANPS) map onto the Big Five personality traits. The investigation of 21 samples with 612 effect sizes available led to a clear picture. As observed in Montag and Panksepp^12^ and in the original ANPS study^7^, links between higher ANGER, SADNESS, FEAR and higher Neuroticism are robust with the highest effect sizes describing the association with SADNESS and FEAR. High SEEKING scores linked to high Openness to Experience. High CARE and low ANGER map onto high Agreeableness, whereas higher PLAY is strongly associated with higher Extraversion.

Considering the effort to measure the expression of primary mammalian emotions with the ANPS versus the derivation of the Big Five from the statistical analysis of descriptive adjectives, the original paper^7^ suggested that the Big Five “represents a human language reconfiguration of underlying primary mammalian affective systems into useful phenotypic descriptive systems”^7^, [p. 67]. However, the risk in the Big Five “lumping” is the potential for clouding the important impact each of these separate emotional forces have in our lives.

In this context, we also mention a moderately strong association in this meta-analysis between higher SEEKING and higher Extraversion, which had been observed/discussed previously, for example in^12,42^. Such a correlation pattern is also in line with the idea that Extraversion is linked to what cognitive neuroscientists would call “reward processing” (for a discussion see^43^), with the SEEKING system being of high relevance as an energetic “Go get it system”. Yet, it is also possible that the Extraversion/SEEKING correlation is derived from Five Factor Model data with adjective-based lexically derived Big Five type assessments showing more modest Extraversion/SEEKING correlations. However, additional studies will be required to answer such questions.

We believe our findings to be remarkable from different perspectives. First of all, different Big Five and ANPS measures have been used in the present work (although preliminary, we see in the present meta-analysis four significantly stronger associations with the NEO-inventories in contrast to the remaining group of Big Five inventories which use adjectives, perhaps reflecting that both the ANPS and the NEO-PI-R inventories work with formulated items). Despite the variety of measures used, the overall pattern of associations carved out strongly supports the idea of robust links between language measures of primary emotional systems and the Big Five with the exception of Conscientiousness, likely being the most cerebral dimension of the Big Five and that has so far only been observed in highly encephalized primates. Nevertheless, our summary at this point is limited by the fact that the SEEKING scale had the highest correlation with Conscientiousness at 0.23 (but still accounted for rather small proportion of variance). Conscientiousness might be seen as a personality trait that is not strongly linked to primary emotional systems, but clearly impacting upon the activity levels of these systems: Higher Conscientiousness might go along with higher top-down control of primary emotional systems.

Second, the present findings are noteworthy, because the investigated samples stem from around the globe, with many samples from Asia (China, Hong Kong, Japan and Eurasian Turkey), but also Europe (France, Germany, Italy, Poland, Spain, Serbia) and the USA. In so far, we believe that the findings observed here are indeed supportive of “a global ancestral neuro-biological effect” as described in^12^ (p. 6). But again, the present meta-analysis does not deal with neuroscientific experimental manipulations, but “only” correlational questionnaire data assessing individual differences in the Big Five personality traits and primary emotional systems. This said, the investigation of the Big Five personality traits in relation to Pankseppian Affective Neuroscience Theory framework seems to shed light on the evolutionary foundations of personality as measured by the Big Five. We mentioned earlier that primary emotional systems have been homologously observed across the mammalian brain, as they endow mammals with tools for survival. Still, individual differences in primary emotional systems exist – meaningfully covarying with (four out of the) Big Five as presented in this work—probably best understood with the concept of fluctuation selection. In other words: From a general psychologist’s perspective it makes evolutionary sense that all mammals are endowed with primary emotional systems, but from a personality psychologist’s perspective this also makes sense, because in some niches different operating levels of a primary emotional system might be favorable.

Third, the overlap between Panksepp’s primary emotional systems and the Big Five provide researchers a route to study the biological basis of the Big Five. Whereas the Big Five personality traits have been merely described using a lexical approach (and basing on this formulated items such as with the NEO-inventories) and therefore do not guide researchers towards an understanding of their biological bases, the substantial overlap of primary emotional systems and the Big Five—as described in this paper—show that neuroanatomical / neurochemical knowledge about the primary emotional systems might also guide neuroscientific studies on the Big Five (how to apply AN theory for such a research endeavor, please see^42^). But again, the present research must be supplemented by neuroscientific techniques and needs further evidence that the ANPS adequately captures the neurobiology of the primary emotional systems as described by Panksepp in much detail^9^; see also the recent review paper on the ANPS by Montag et al.^44^. As some primary emotional systems are not uniquely associated with one Big Five personality trait, (e.g. ANGER negatively links to Agreeableness and positively to Neuroticism), it will be also of high interest to understand the brain mechanisms leading to multiple primary emotional brain systems being combined in different personality traits—likely via different excitatory or inhibitory effect levels.

What are the future directions? Only recently, researchers became interested in investigating the ANPS and the Big Five also on the facets level^16,26^. For instance, Montag et al.^16^ observed a particular strong association between Neuroticism’s facet anger hostility and ANGER (0.69). Another example: Regarding the primary emotional system of CARE relations with Agreeableness facets tender-mindedness (0.48) and altruism (0.46) were pronounced. As only very few investigations have been carried out in this area, many more studies need to be published with such a research focus to understand whether primary emotional systems based on the background of Pankseppian AN theory are multifaceted and if so linked to Big Five facets. Although the ANPS has been translated into many languages already (see introduction), we want to encourage researchers to translate the ANPS also to further languages and ideally validate it with the Big Five (as presented in this work). Finally, other personality theories or extensions of the Big Five model need to be more strongly linked to AN theory. For instance, the work by Knezevic and colleagues^45^ investigated the ANPS in the context of the HEXACO model, thereby also investigating how Honesty/Humility is linked to individual differences in primary emotional systems.

Beyond that most studies in the field are correlational and it would be highly interesting to see longitudinal works assessing covariations between primary emotional systems and the Big Five across the life span (at best also supplemented by neuroscientific data). Concluding, the present meta-analysis provides solid evidence for the idea that primary emotional systems as measured by the ANPS are meaningfully linked with the Big Five personality traits. Moreover, studying individual differences of the Big Five personality traits using a Pankseppian Affective Neuroscience Theory framework also provides researchers an evolutionary approach to understand the lexically derived Big Five personality constructs.

## Supplementary Information

Supplementary Information 1.

Supplementary Information 2.
