# Supplementary material for: A meta-analysis on individual differences in primary emotional systems and Big Five personality traits
Source: Sci Rep. 2021 Apr 2;11:7453. doi: 10.1038/s41598-021-84366-8 (PMC8018956; doi:10.1038/s41598-021-84366-8)

Figure S1. Funnel Plot of Fisher’s Z and Standard Error of the Correlation between ANGER and Agreeableness


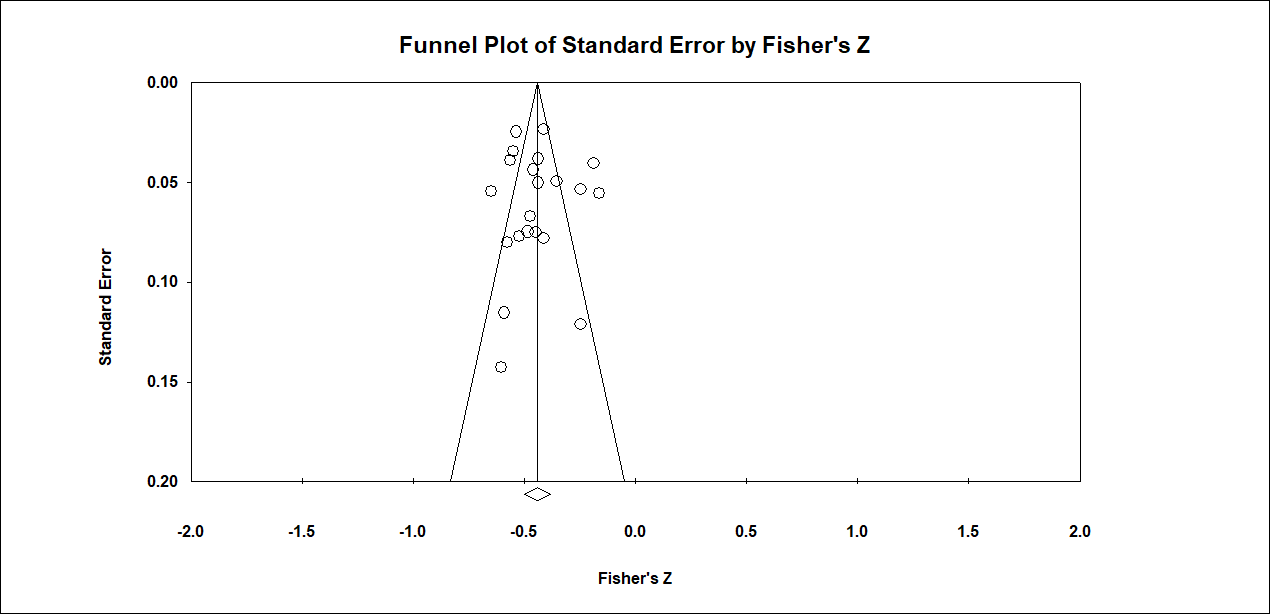


Figure S2. Funnel Plot of Fisher’s Z and Standard Error of the Correlation between ANGER and Conscientiousness


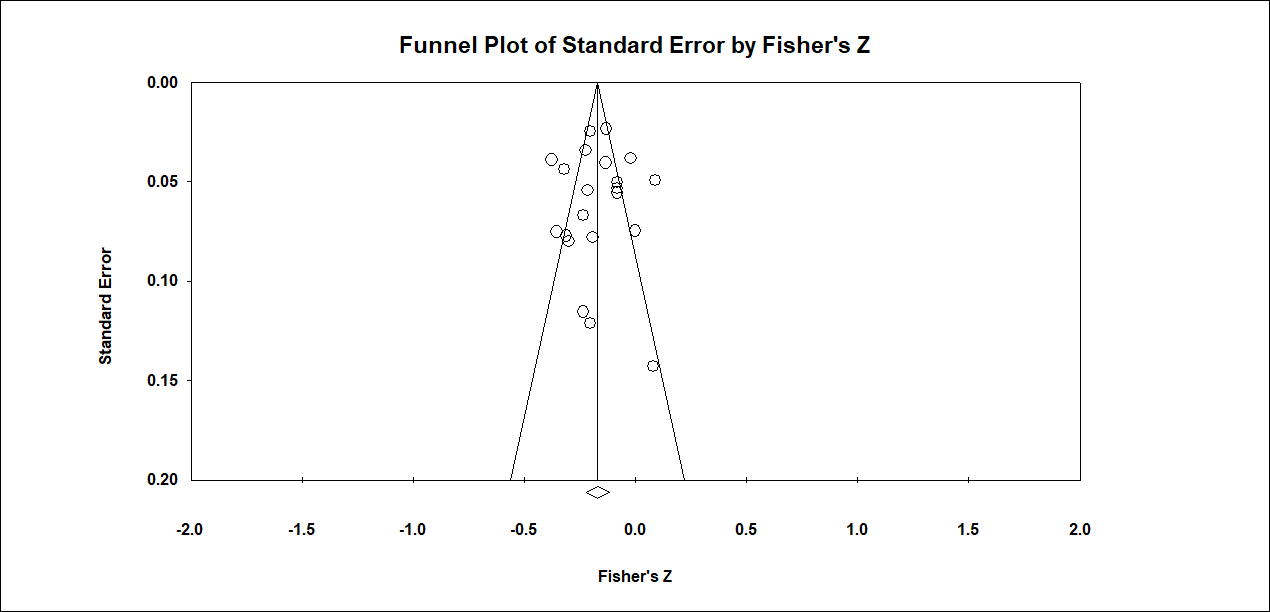


Figure S3. Funnel Plot of Fisher’s Z and Standard Error of the Correlation between ANGER and Extraversion


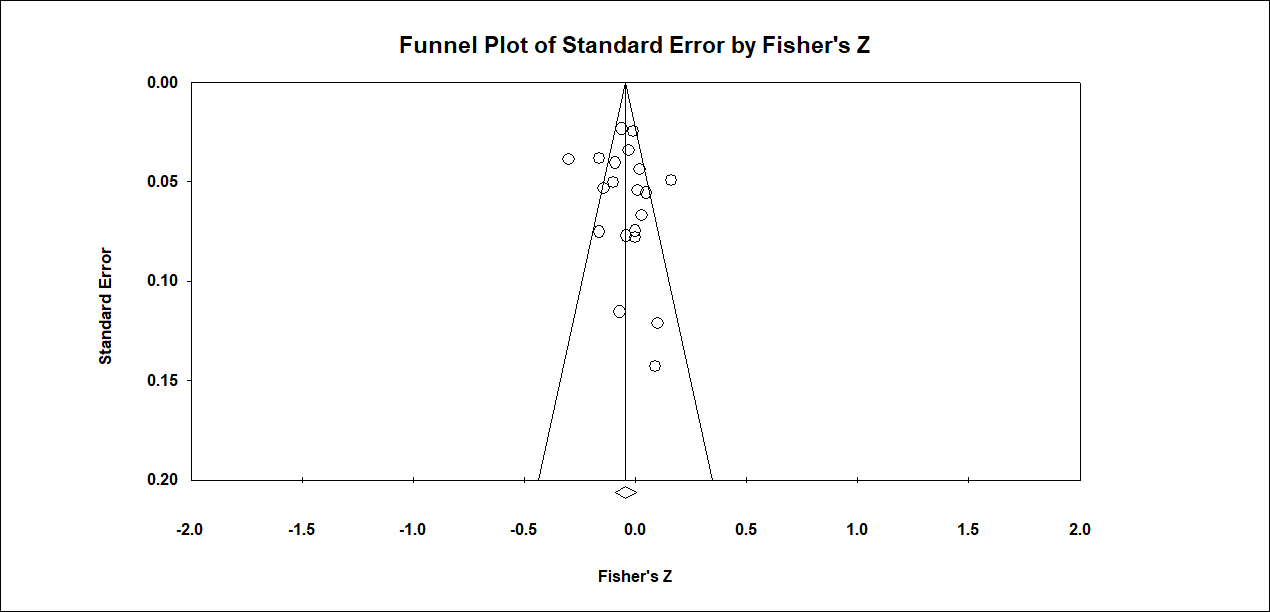


Figure S4. Funnel Plot of Fisher’s Z and Standard Error of the Correlation between ANGER and Neuroticism


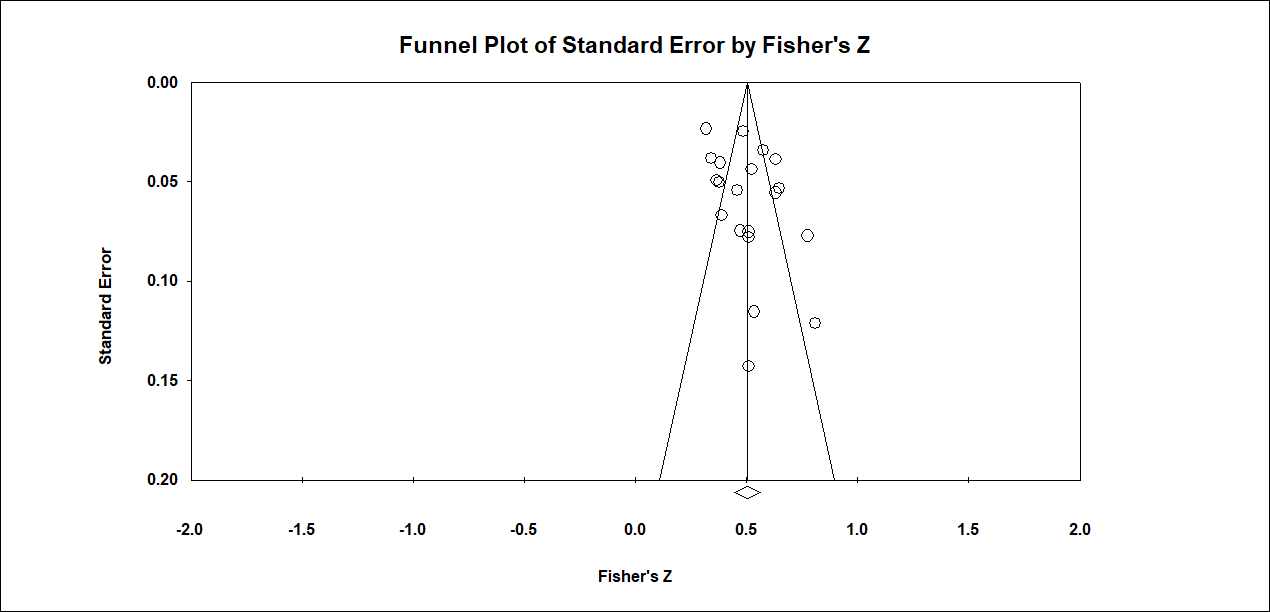


Figure S5. Funnel Plot of Fisher’s Z and Standard Error of the Correlation between ANGER and Openness


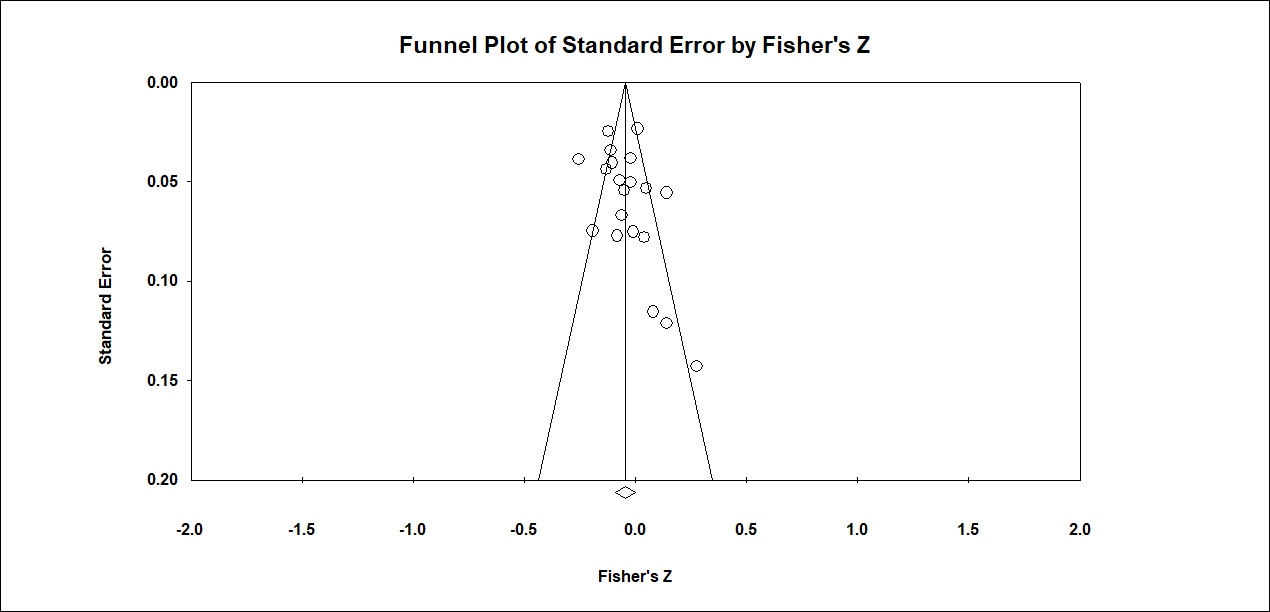


Figure S6. Funnel Plot of Fisher’s Z and Standard Error of the Correlation between CARE and Agreableness


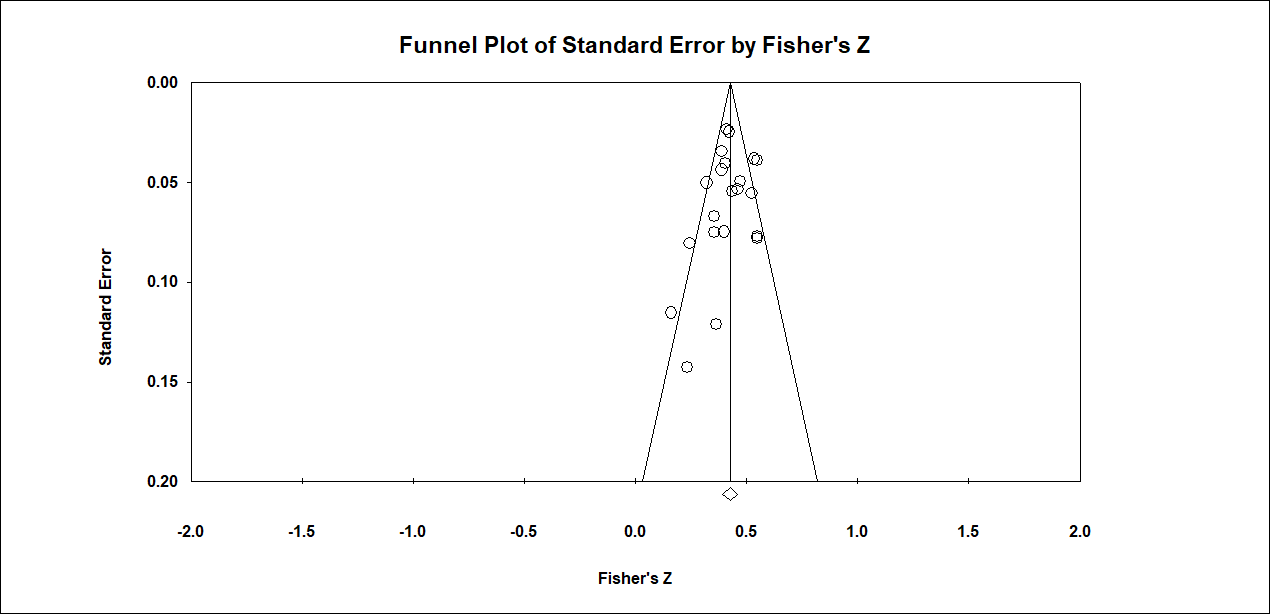


Figure S7. Funnel Plot of Fisher’s Z and Standard Error of the Correlation between CARE and Conscientiousness


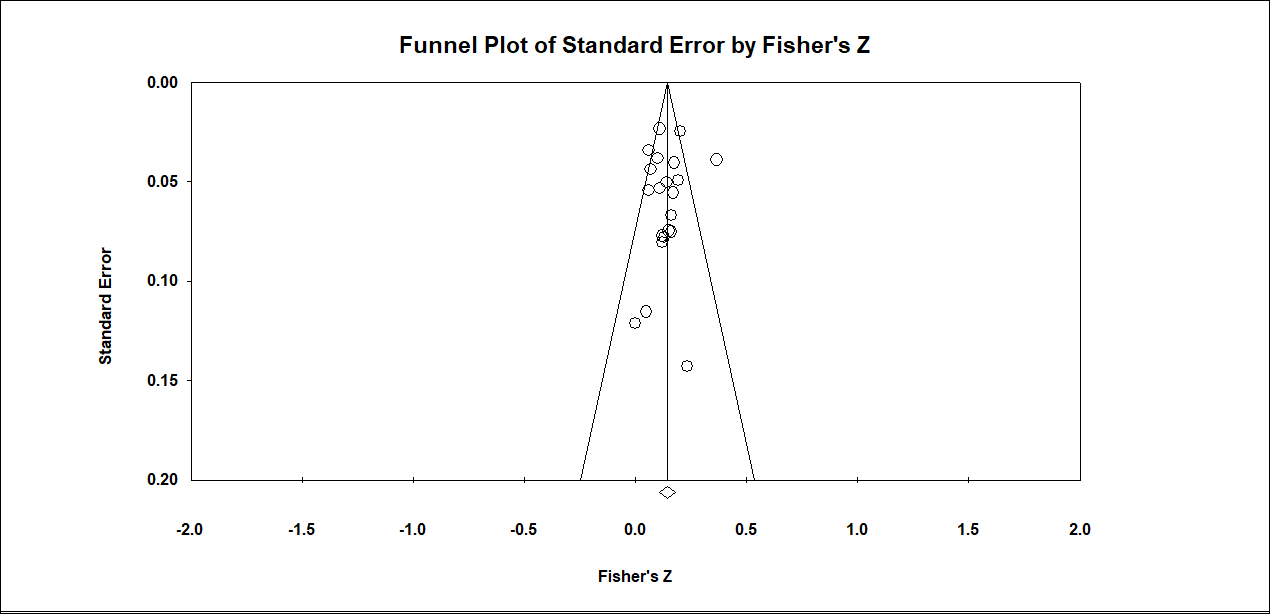


Figure S8. Funnel Plot of Fisher’s Z and Standard Error of the Correlation between CARE and Extraversion


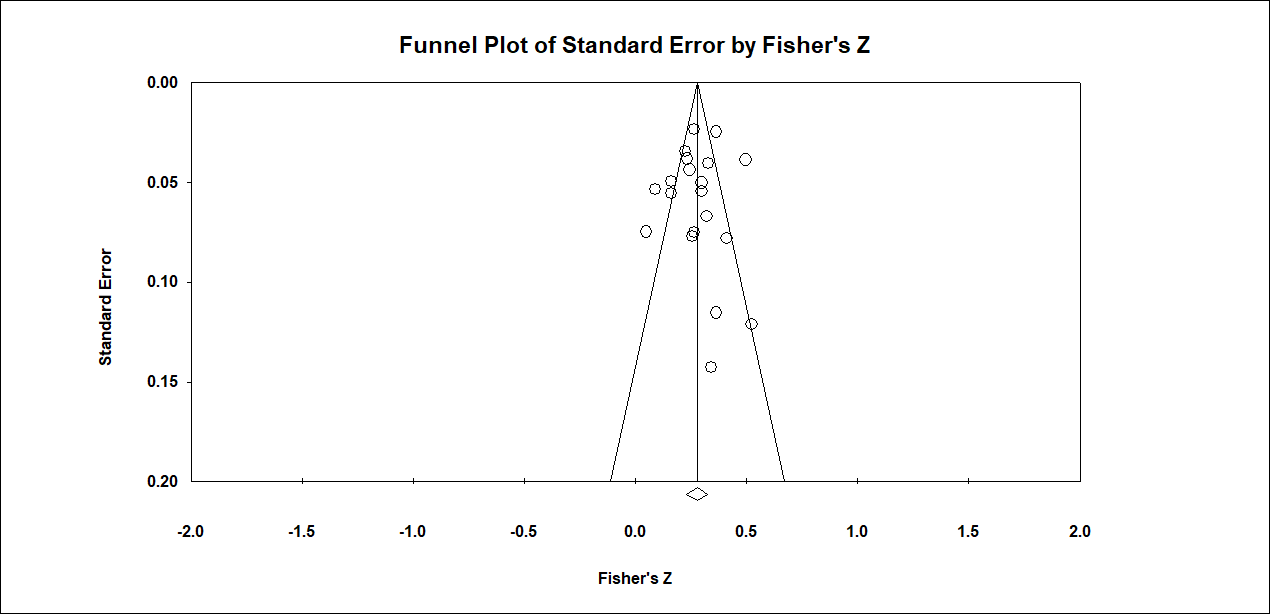


Figure S9. Funnel Plot of Fisher’s Z and Standard Error of the Correlation between CARE and Neuroticism


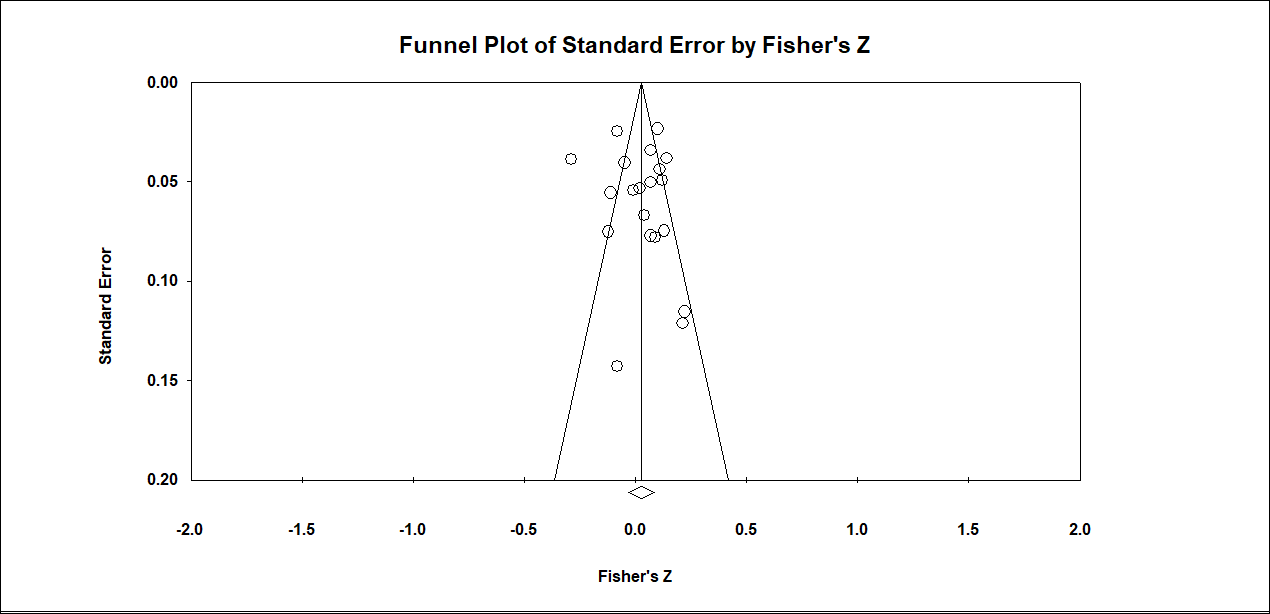


Figure S10. Funnel Plot of Fisher’s Z and Standard Error of the Correlation between CARE and Openness


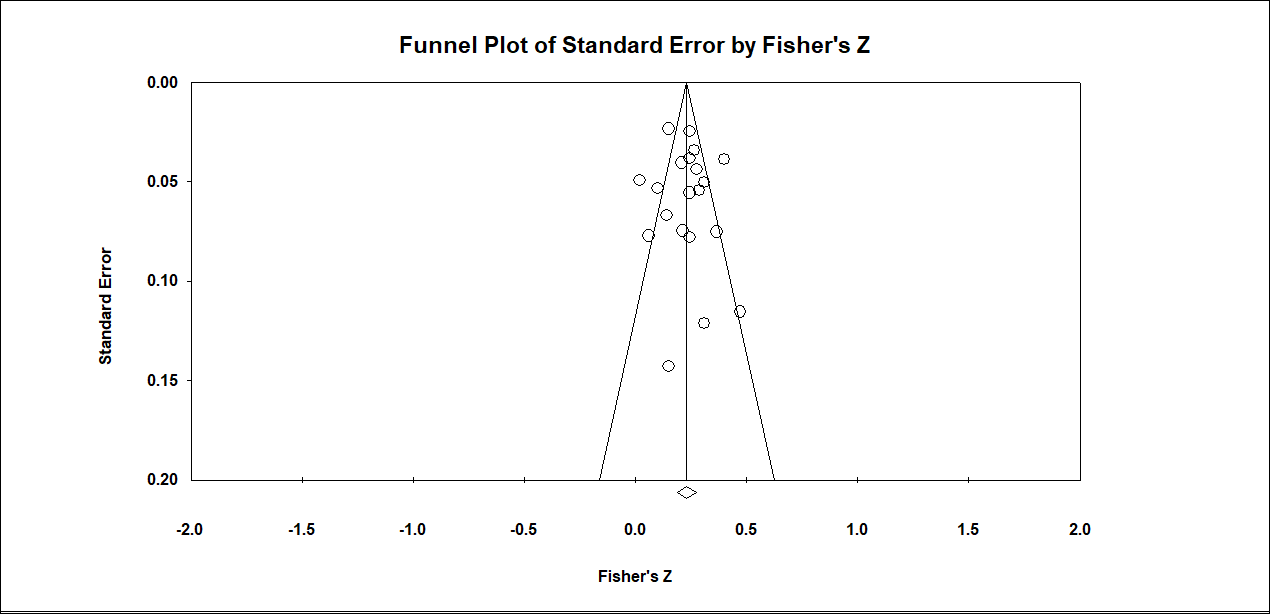


Figure S11. Funnel Plot of Fisher’s Z and Standard Error of the Correlation between FEAR and Agreeableness


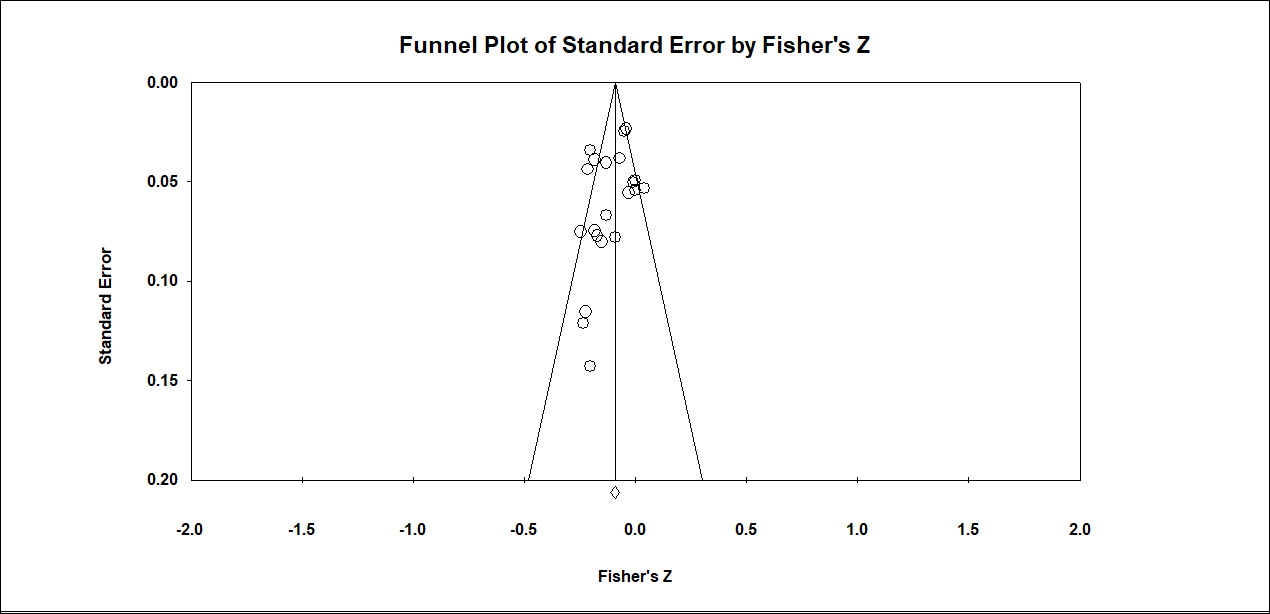


Figure S12. Funnel Plot of Fisher’s Z and Standard Error of the Correlation between FEAR and Conscientiousness


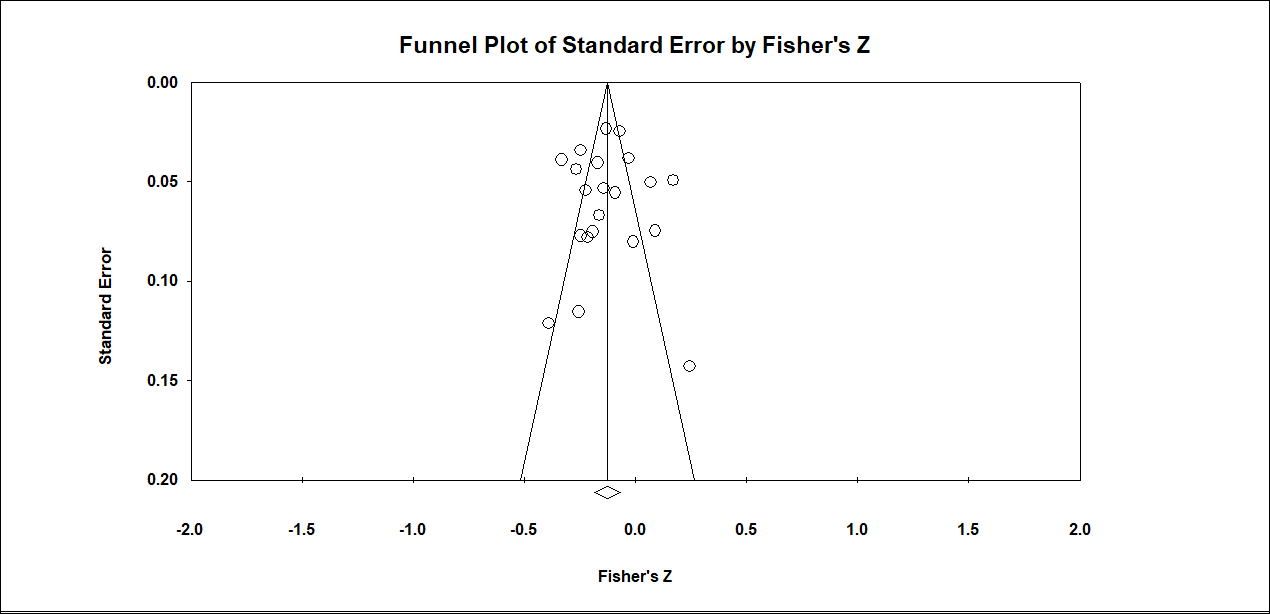


Figure S13. Funnel Plot of Fisher’s Z and Standard Error of the Correlation between FEAR and Extraversion


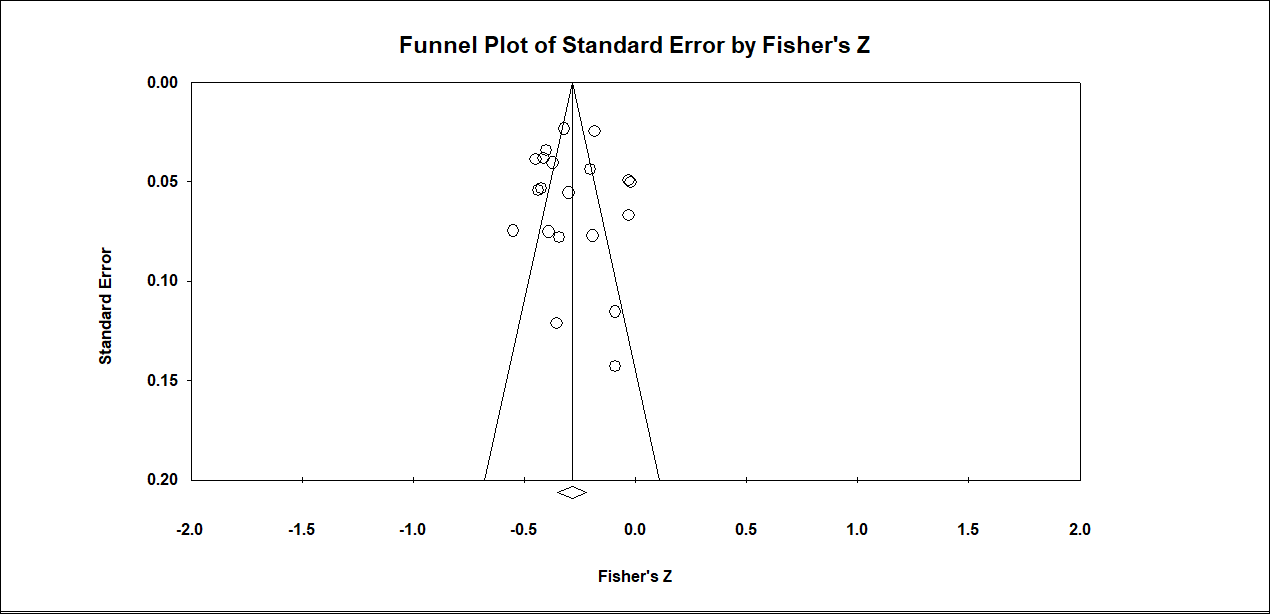


Figure S14. Funnel Plot of Fisher’s Z and Standard Error of the Correlation between FEAR and Neuroticism


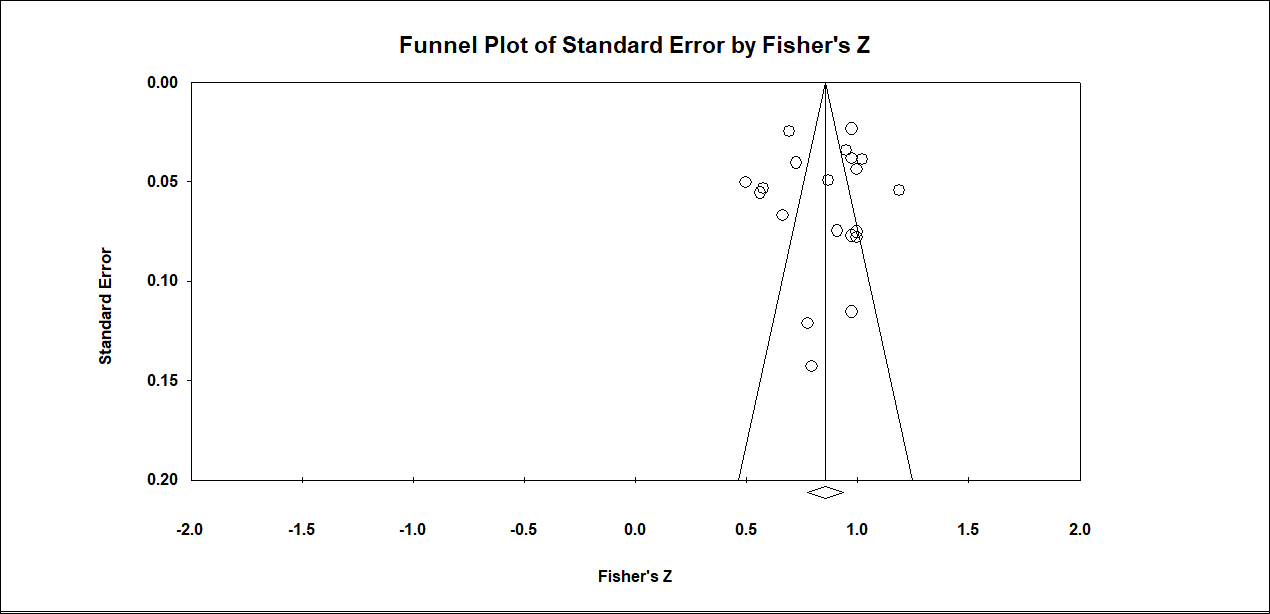


Figure S15. Funnel Plot of Fisher’s Z and Standard Error of the Correlation between FEAR and Openness


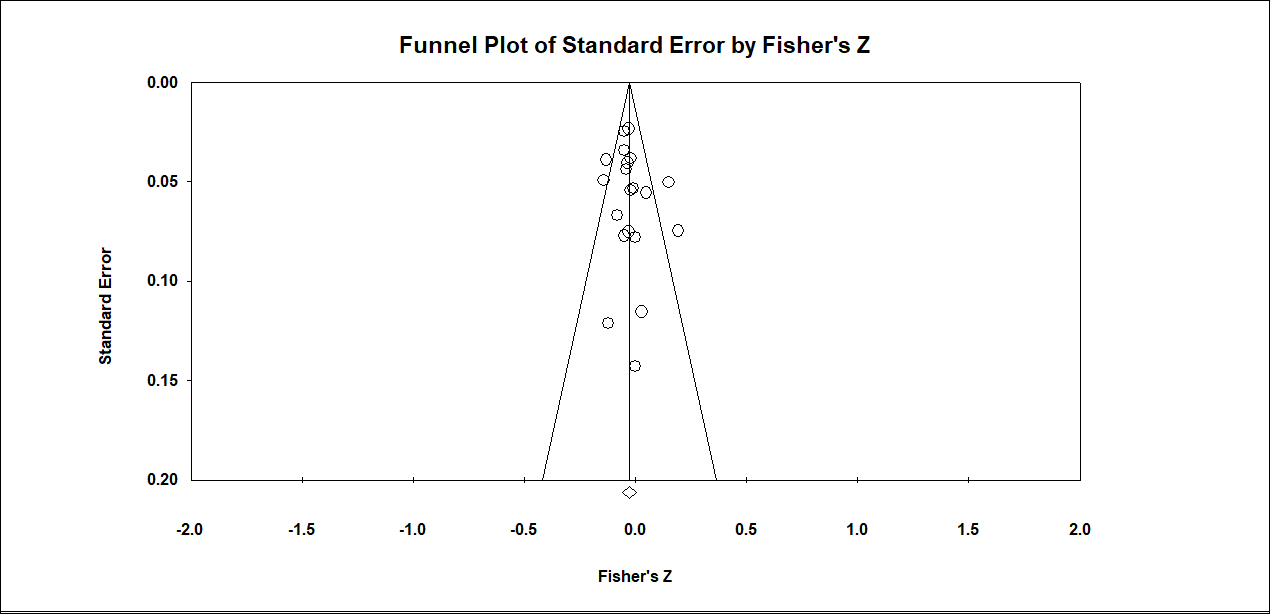


Figure S16. Funnel Plot of Fisher’s Z and Standard Error of the Correlation between PLAYING and Agreeableness


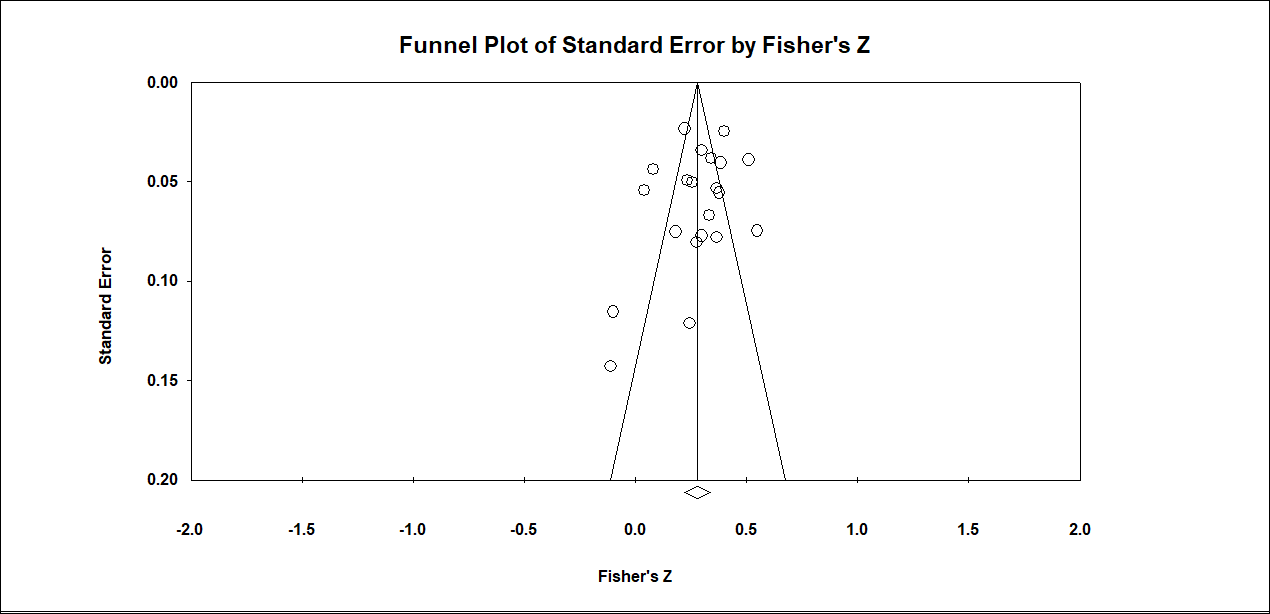


Figure S17. Funnel Plot of Fisher’s Z and Standard Error of the Correlation between PLAYING and Conscientiousness


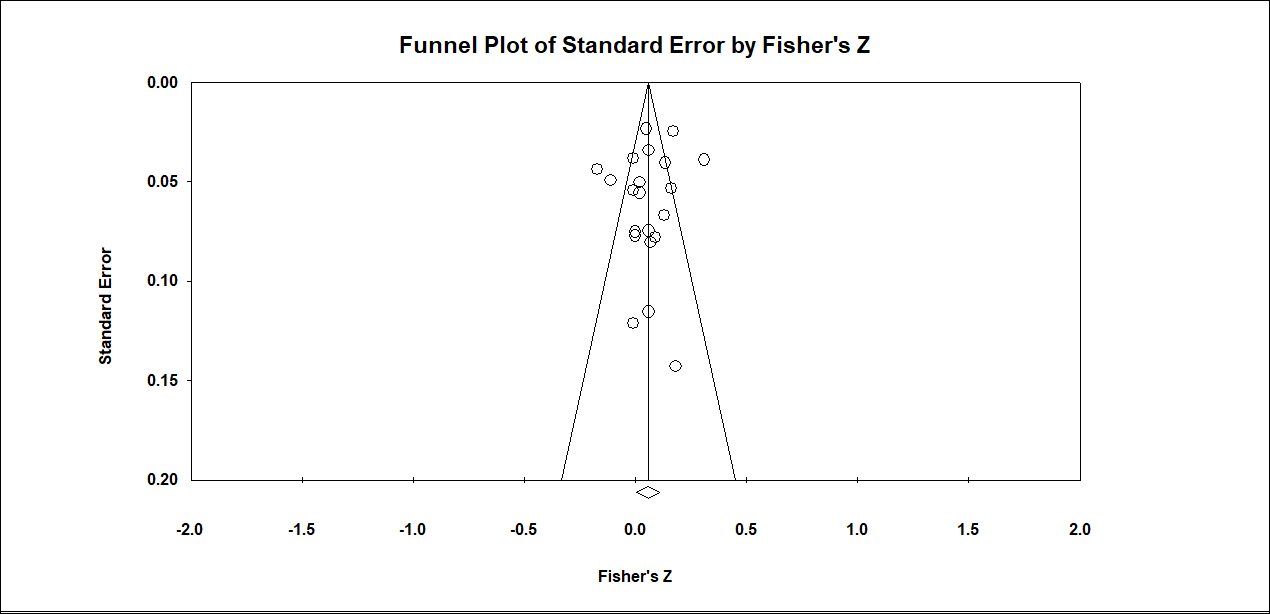


Figure S18. Funnel Plot of Fisher’s Z and Standard Error of the Correlation between PLAYING and Extraversion


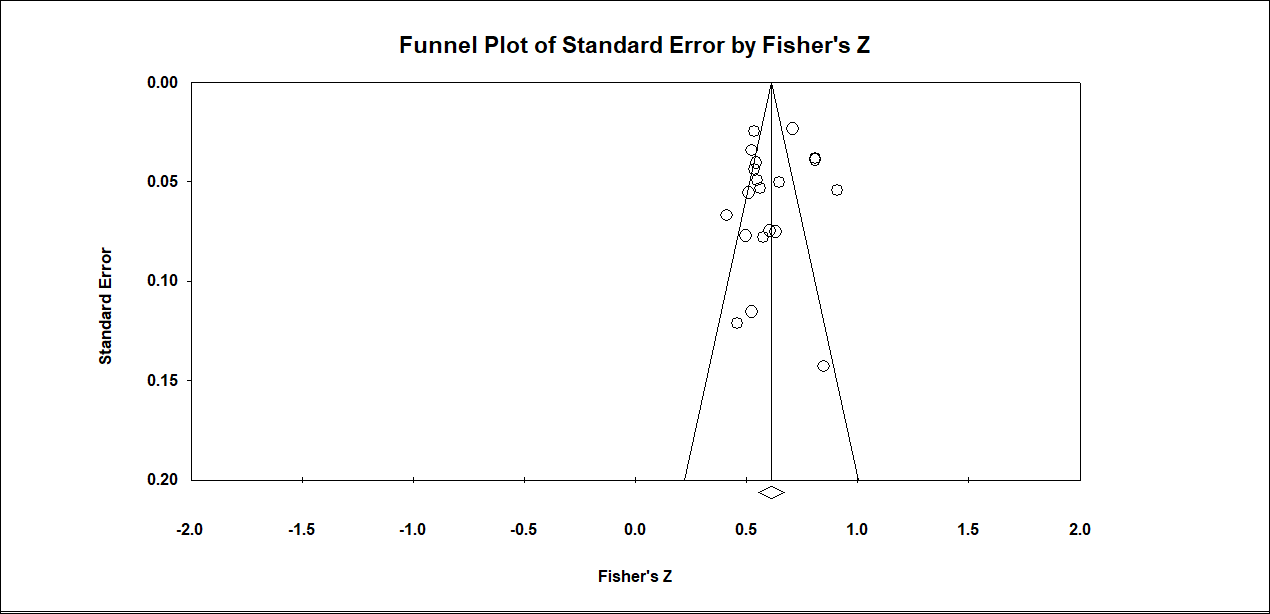


Figure S19. Funnel Plot of Fisher’s Z and Standard Error of the Correlation between PLAYING and Neuroticism


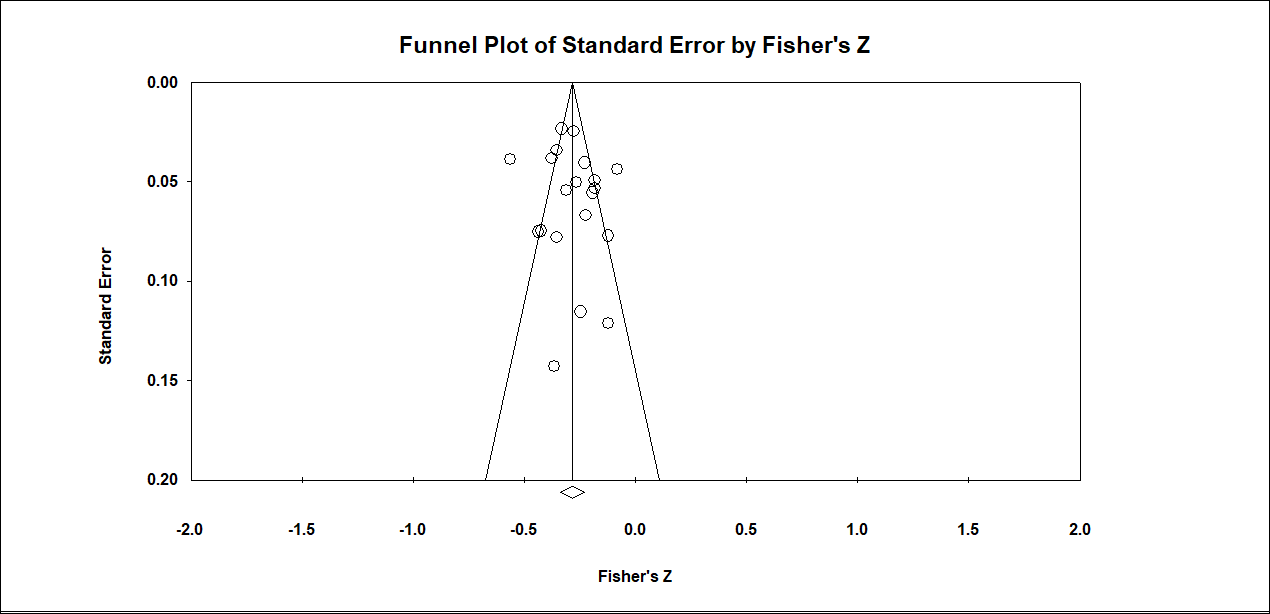


Figure S20. Funnel Plot of Fisher’s Z and Standard Error of the Correlation between PLAYING and Openness


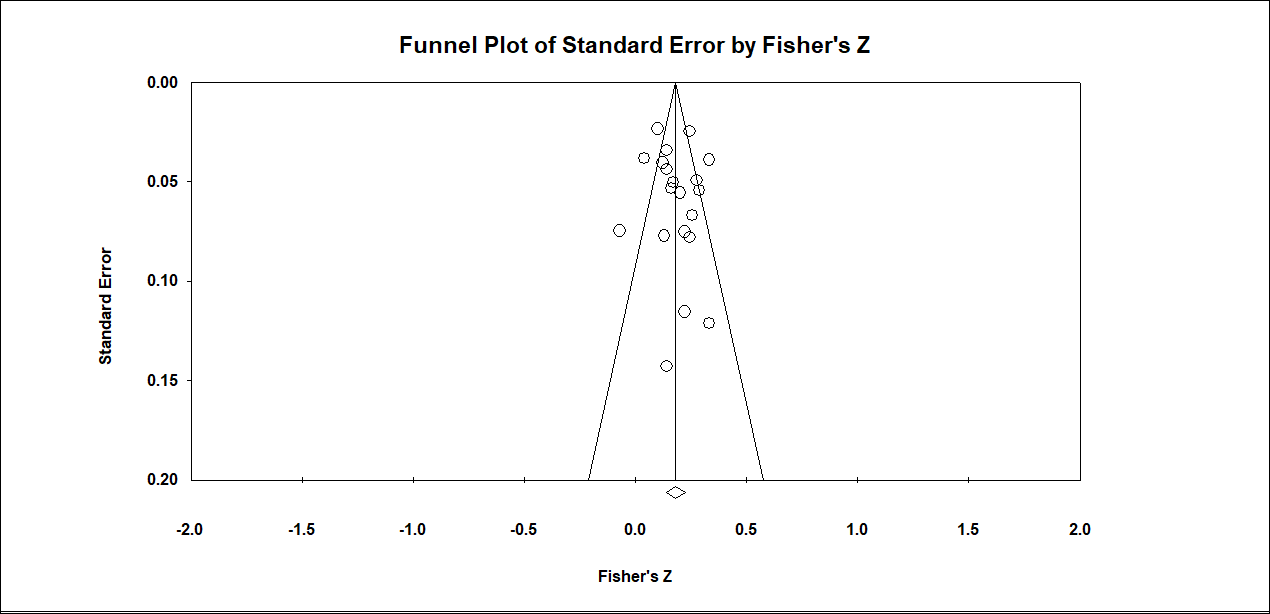


Figure S21. Funnel Plot of Fisher’s Z and Standard Error of the Correlation between SADNESS and Agreeableness


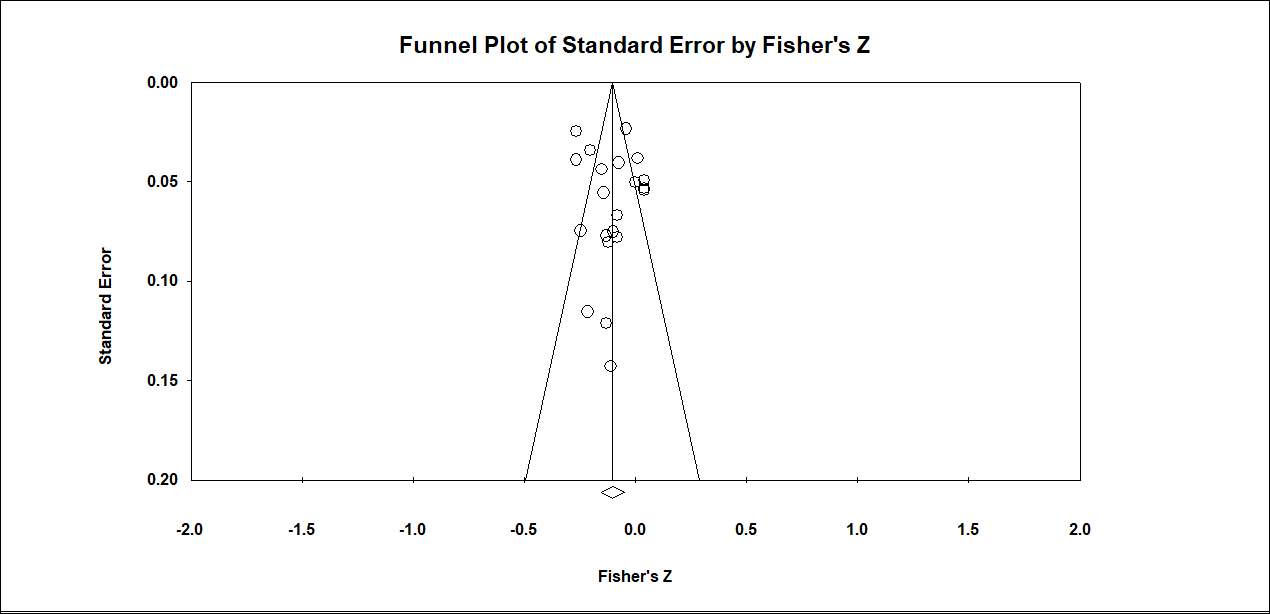


Figure S22. Funnel Plot of Fisher’s Z and Standard Error of the Correlation between SADNESS and Conscientiousness


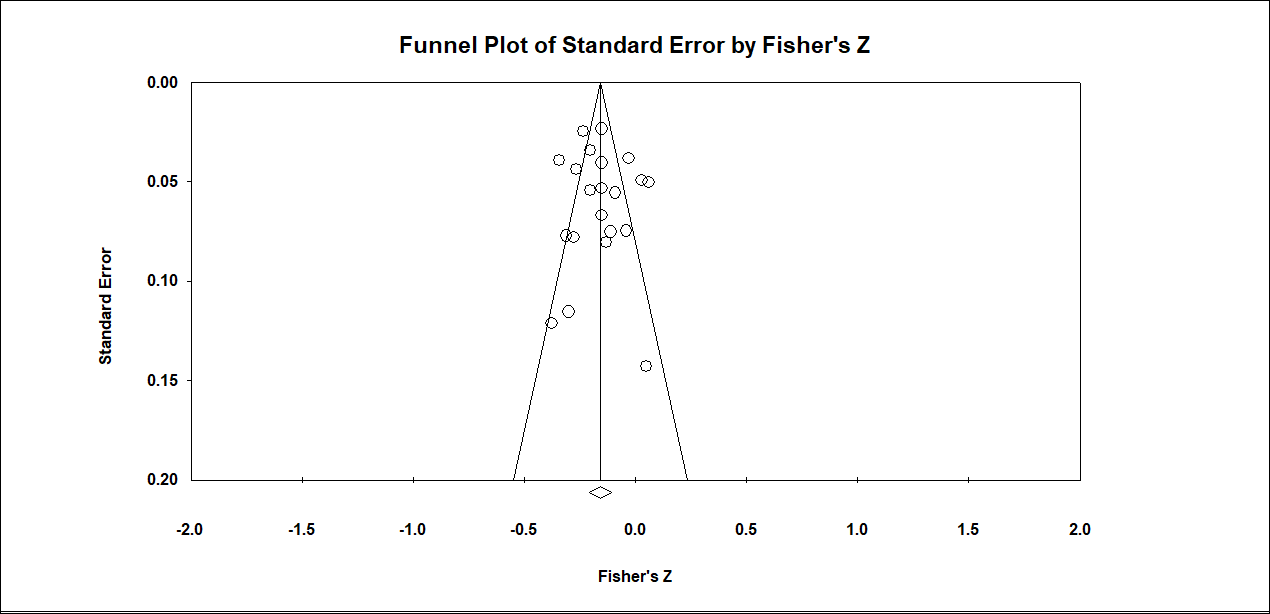


Figure S23. Funnel Plot of Fisher’s Z and Standard Error of the Correlation between SADNESS and Extraversion


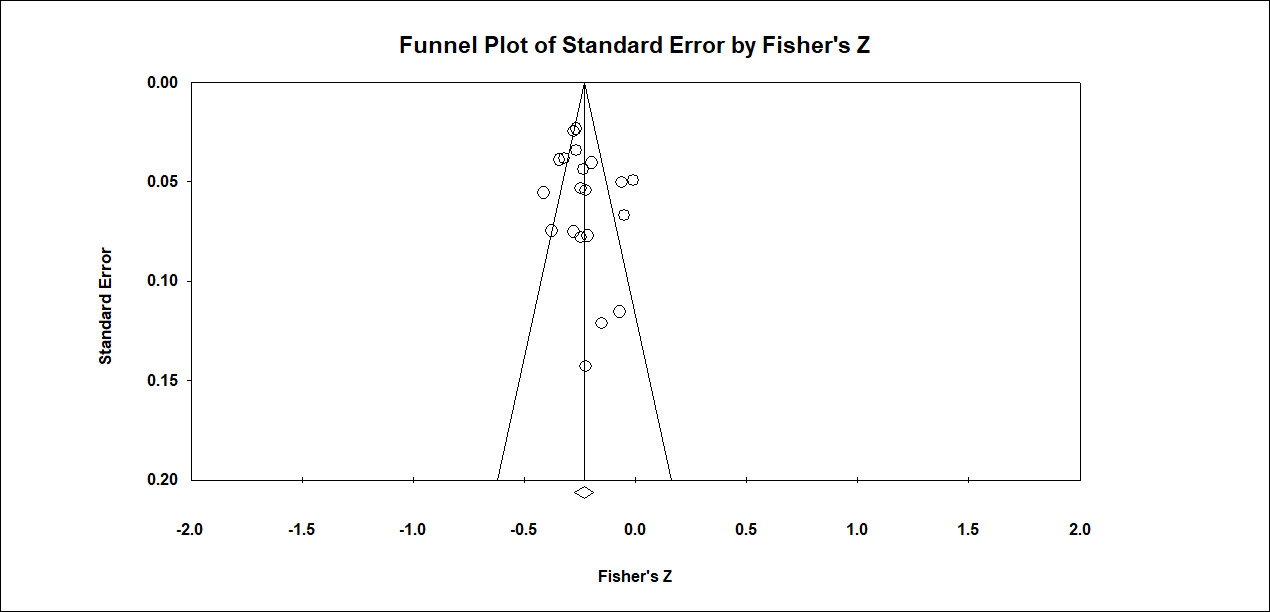


Figure S24. Funnel Plot of Fisher’s Z and Standard Error of the Correlation between SADNESS and Neuroticism


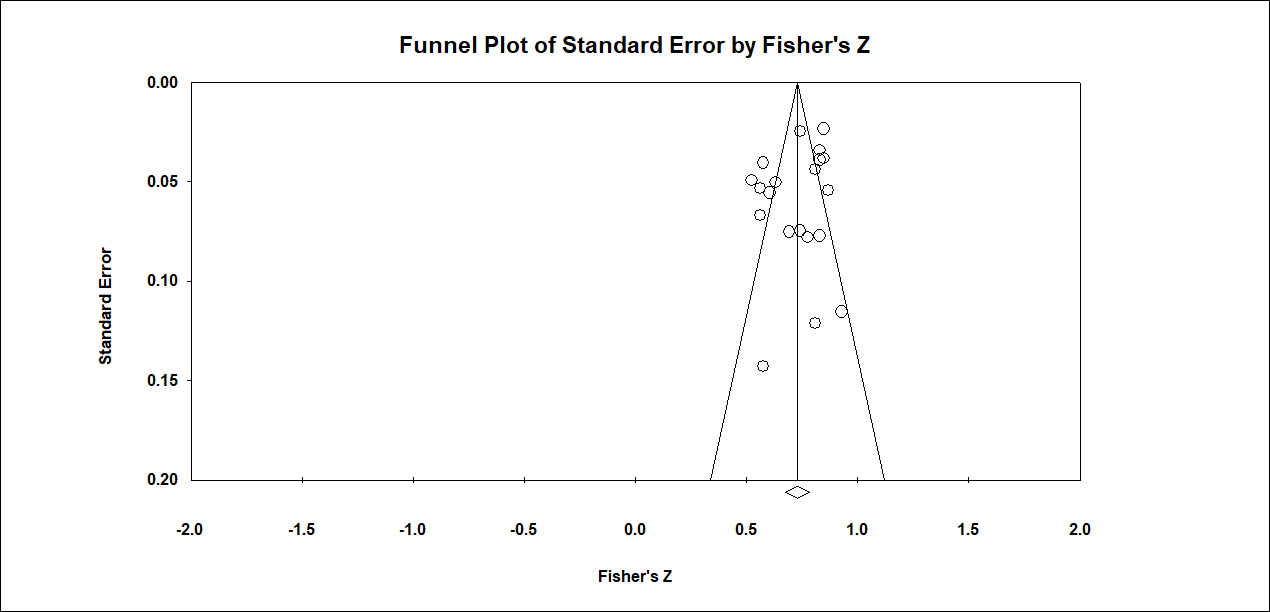


Figure S25. Funnel Plot of Fisher’s Z and Standard Error of the Correlation between SADNESS and Openness


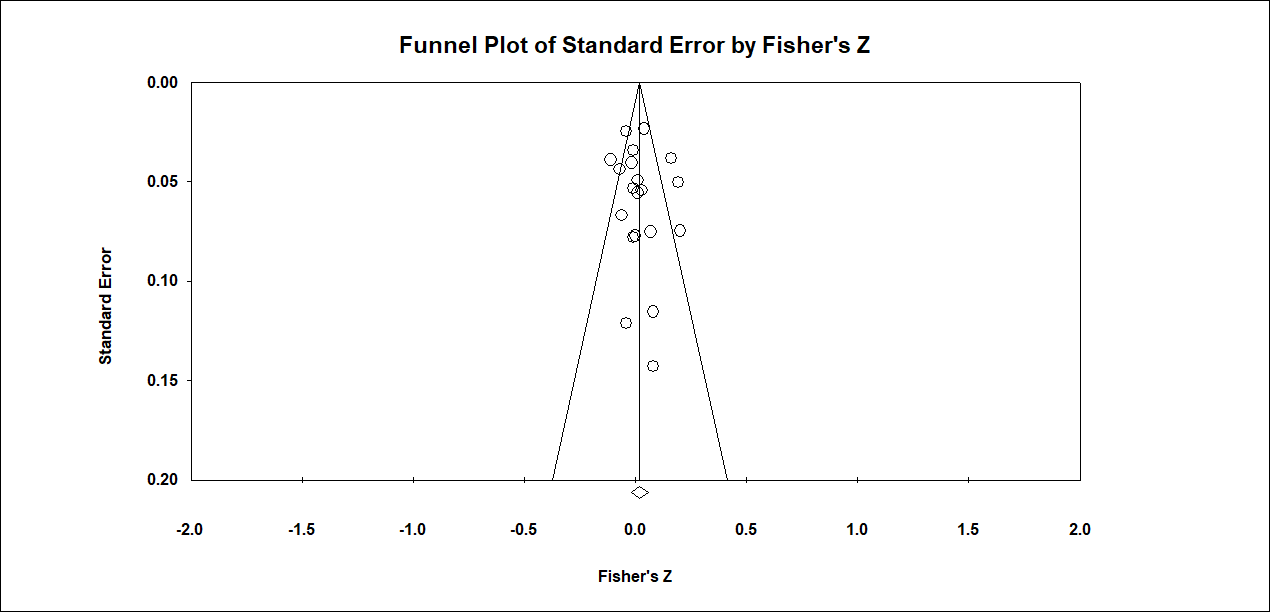


Figure S26. Funnel Plot of Fisher’s Z and Standard Error of the Correlation between SEEKING and Agreeableness


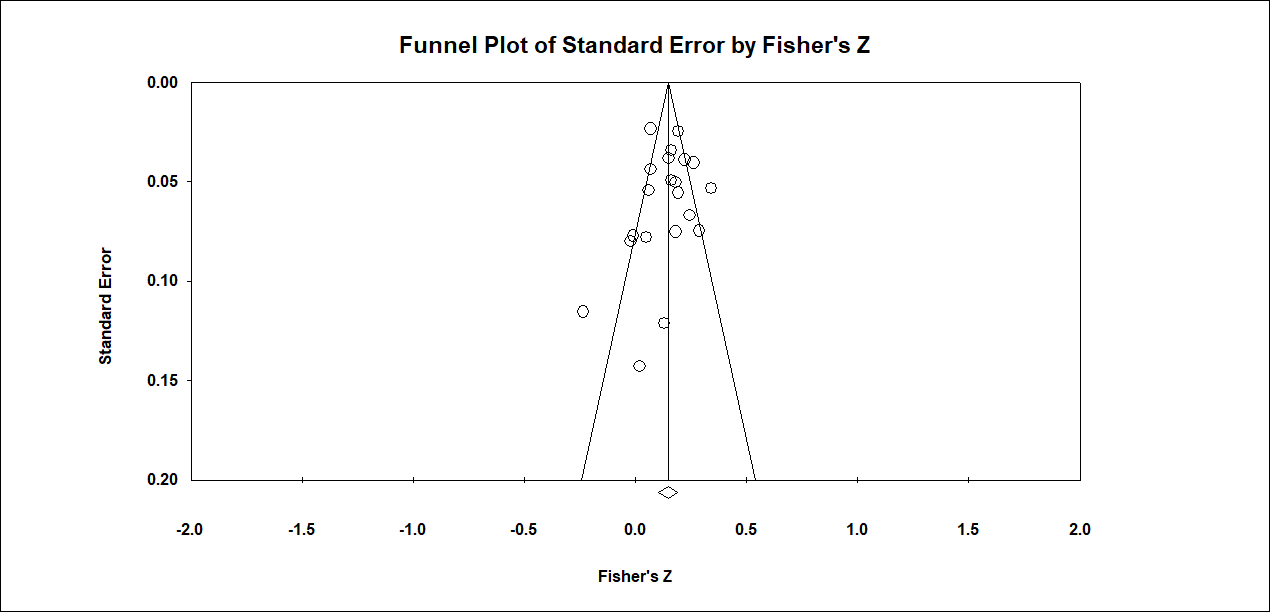


Figure S27. Funnel Plot of Fisher’s Z and Standard Error of the Correlation between SEEKING and Conscientiousness


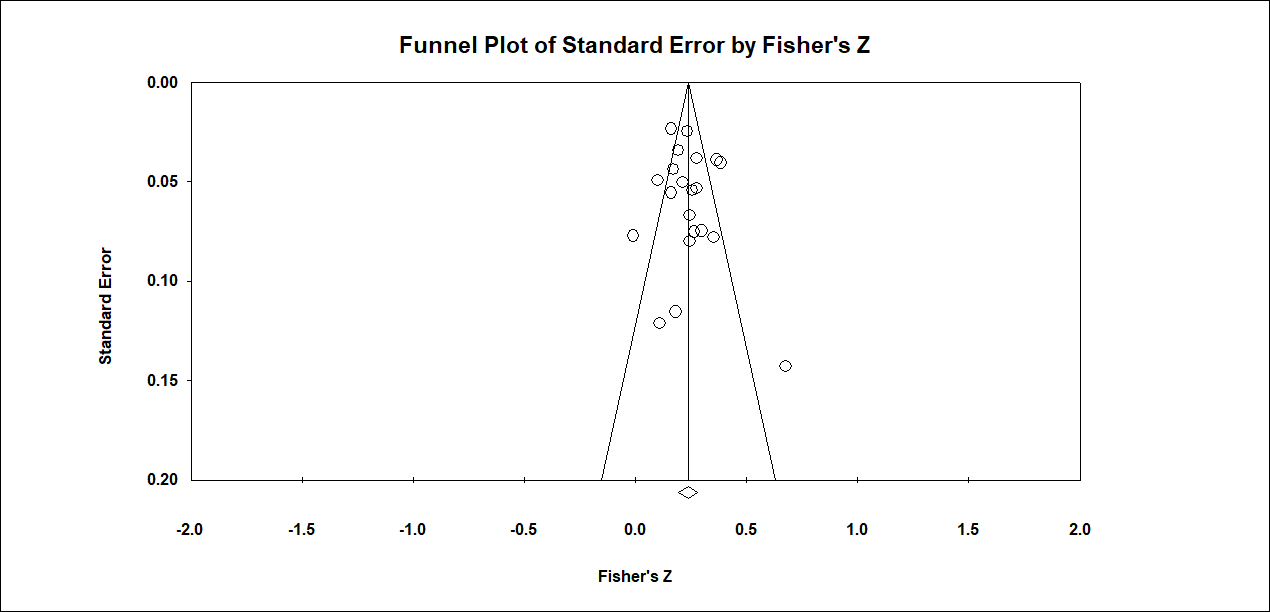


Figure S28. Funnel Plot of Fisher’s Z and Standard Error of the Correlation between SEEKING and Extraversion


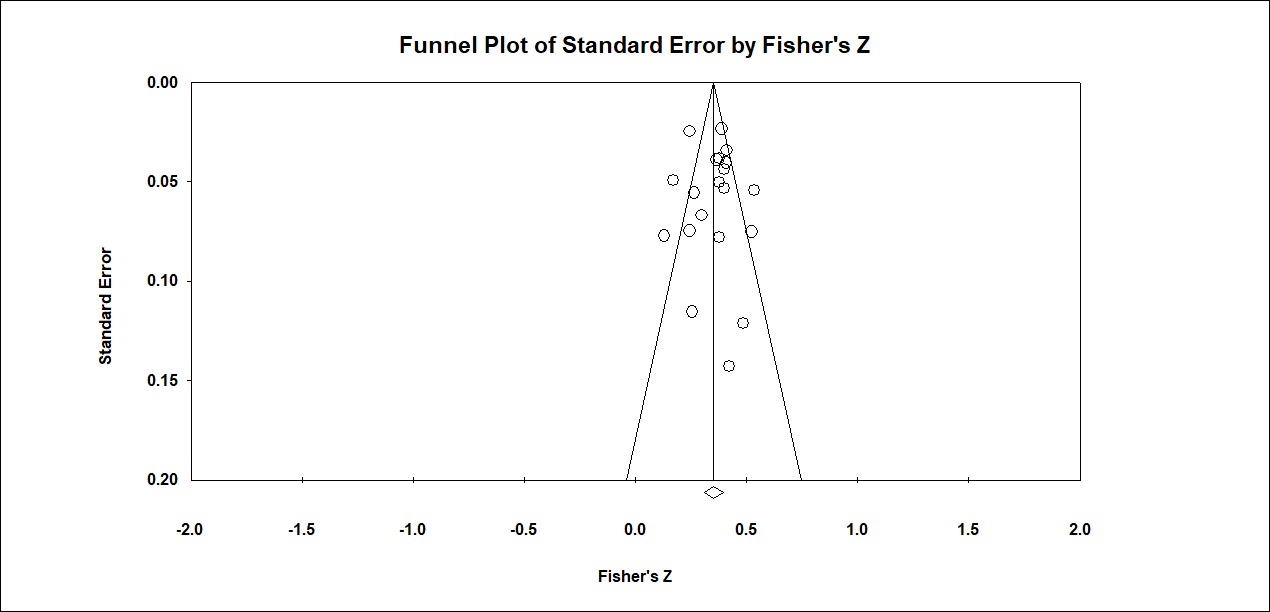


Figure S29. Funnel Plot of Fisher’s Z and Standard Error of the Correlation between SEEKING and Neuroticism


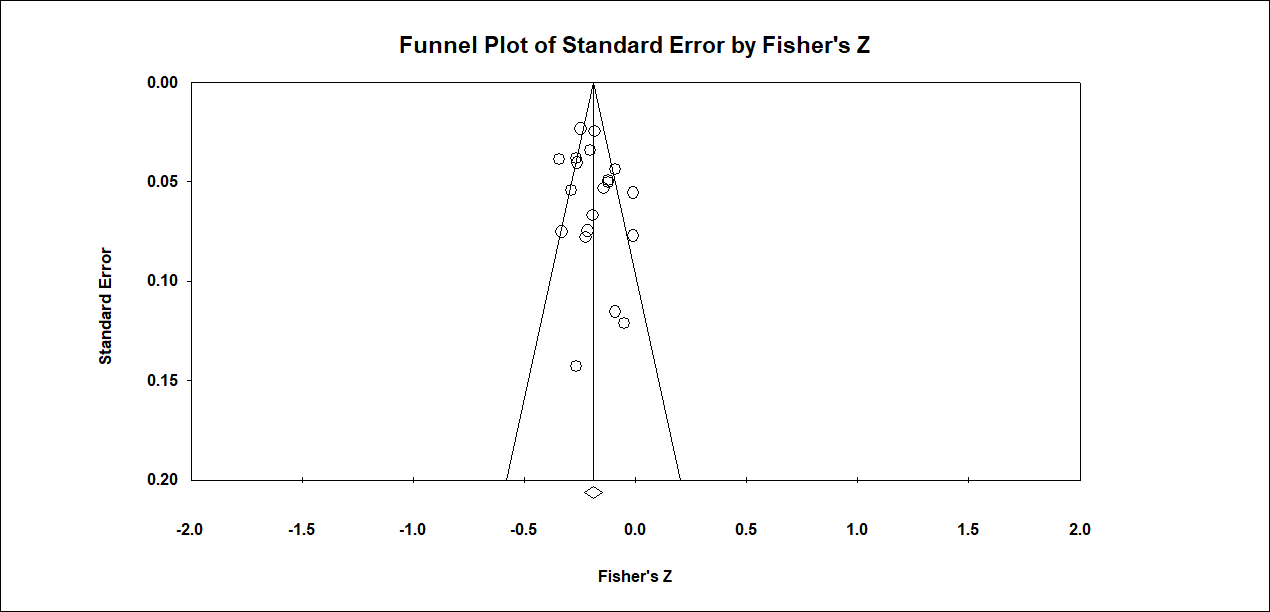


Figure S30. Funnel Plot of Fisher’s Z and Standard Error of the Correlation between SEEKING and Openness


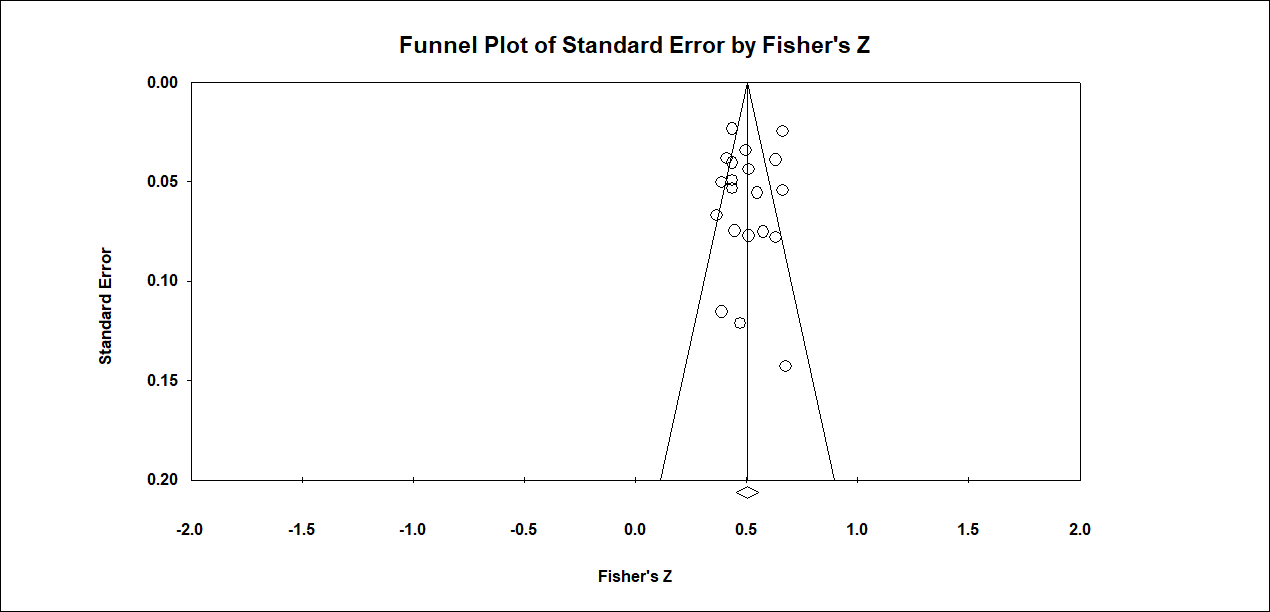

Supplement: Supplementary file 1 — Supplementary Information 1. [file 41598_2021_84366_MOESM1_ESM.docx]
